# Gain-adaptive STAR-RIS assisted vehicular NOMA with transmit antenna selection

**DOI:** 10.1038/s41598-026-58445-7

**Published:** 2026-06-29

**Authors:** Shady M. Ibraheem, Mona Shokair, Mohamed E. Nasr, Nancy Alshaer

**Affiliations:** 1 Electronics and Electrical Communications Department, Faculty of Engineering, Tanta University, Tanta, 31527 Egypt; 2 Telecom Egypt, Cairo, Egypt; 3 Electronics and Electrical Communications Department, Faculty of Electronic Engineering, Menoufia University, Menoufia, Egypt; 4 Electronics and Electrical Communications Department, Faculty of Engineering, October 6 University, 6th of October City, Egypt

**Keywords:** Non-orthogonal multiple access, Reconfigurable intelligent surface, Outage probability, Ergodic sum rate, Diversity gain, Engineering, Mathematics and computing

## Abstract

This paper proposes a dynamic gain-adaptive scheme for simultaneously transmitting and reflecting reconfigurable intelligent surface (STAR-RIS) based non-orthogonal multiple access (NOMA) networks (termed as ASRN) with a transmit antenna selection technique at the base station to serve two vehicular users. Unlike conventional fixed-ranking NOMA, ASRN dynamically allocates power and assigns successive interference cancellation order to users based on their channel conditions. Firstly, we derive closed-form expressions for the system outage probability, asymptotic outage behavior, and diversity order, revealing that the proposed scheme achieves a diversity gain scaling with the number of STAR-RIS elements. Then, a greedy algorithm is further proposed to jointly optimize antenna selection and NOMA users’ pairing. Secondly, we introduce a key performance metric (termed as the ergodic sum rate (ESR)) and define the multiple averaging ergodic sum rate (MA-ESR) to evaluate the effectiveness of ASRN scheme in delay-tolerant networking approach, when compared to other schemes. Monte Carlo simulations demonstrate that ASRN significantly outperforms dynamic gain-adaptive STAR-RIS OMA conventional STAR-RIS NOMA/OMA, and decode-and-forward relaying schemes, offering superior outage performance, coding gains, ESR performance and MA-ESR performance, particularly in vehicular environments.

## Introduction

Reconfigurable intelligent surface (RIS) technology has been envisioned as an innovative, simple, and cost-effective promising technology for next-generation wireless networks. RIS consists of abundant passive meta-elements that can precisely reconfigure the amplitude and phase shift of the incident electromagnetic signal to create a controllable wireless propagation environment^1,2^. However, conventional reflecting-only RIS provides a *half-plane* coverage area, which greatly restricts its flexibility and geometrical applications. The concept of simultaneously transmitting and reflecting RIS (STAR-RIS) has been considered to provide a *full-plane* coverage area by splitting the incident signal into transmitted and reflected signals on both sides of the meta-elements and thus allowing a more enhanced radio environment^3,4^.

On the other hand, non-orthogonal multiple access (NOMA) has been considered an innovative technology to support massive connectivity, higher spectral efficiency, and better user fairness^5^. NOMA allows multiple users to share the same resource (time/frequency/code) while receivers can extract their individual signals by the aid of successive interference cancellation (SIC) technique. Cooperative NOMA is an effective approach to extend the coverage area and the reception quality of the network. In^6,7^, max-min relay selection cooperative NOMA schemes were proposed where users were scheduled according to their quality of service (QoS) requirements. However, in vehicular communications networks (as in public transportation), penetration loss may degrade the QoS requirements and cause network outage^8^. Cooperative relay selection NOMA schemes were proposed in^9,10^ where users were ranked according to their instantaneous channel state information (CSI).

Integrating STAR-RIS with NOMA can significantly improve the spectral efficiency and the sum rate performance of NOMA users. A NOMA downlink model for two-user aided by a STAR-RIS under Rician fading was discussed by the work of^11^, where the base station (BS) sends a superimposed NOMA signal; the STAR-RIS splits the incident signal into transmitted and reflected parts to serve the “distant” user and the “nearby” user, respectively. The authors studied the outage probability and ergodic sum rate performance of STAR-RIS assisted NOMA networks. In^12^, the authors proposed a hybrid active relay and STAR-RIS joint selection system to assist two NOMA users where robust system outage performance can be achieved under different path loss conditions.

Moreover, the authors in^13^ proposed a mode-switching protocol for the STAR-RIS. They also proposed a partitioning algorithm to allocate STAR-RIS elements to different users to maximize sum-rate while satisfying QoS constraints. They derived closed-form expressions for the outage probability, analyzed system sum-rate (ergodic or average rate) and compared with conventional NOMA and OMA. In^14^, ergodic rates of STAR-RIS aided NOMA systems over Nakagami-m fading channels were investigated for cell-edge users without Line-of-Sight links from BS. Closed-form expressions of ergodic rates were derived for NOMA users by fitting the composite channel gain to a gamma distribution. Numerical results showed that the STAR-RIS aided NOMA system performs better than the RIS aided NOMA system. The framework of^15^ proposed a low-complexity mode switching design using channel angle information in STAR-RIS aided multi-antenna NOMA systems. The study in^16^ analyzed a STAR-RIS-assisted NOMA for full-duplex communication system and derived closed-form ergodic rate expressions for downlink, uplink, and bidirectional scenarios.

Recent advancements in STAR-RIS technology have introduced active, simultaneously transmitting and reflecting surfaces (ASTARS), which overcome the multiplicative path-loss limitation of passive STAR-RIS by incorporating amplification. In^17^, the authors investigated ASTARS-assisted NOMA networks. They demonstrated that active elements can significantly enhance coverage and spectral efficiency compared to passive counterparts, particularly in scenarios with severe path-loss. Furthermore, STAR-RIS has been explored for secure communications, where^18^ proposed ASTARS-aided NOMA covert communication networks to ensure secure transmission while maintaining a low probability of detection by potential eavesdroppers. The integration of machine learning with STAR-RIS-NOMA systems has also gained attention, as evidenced by^19^, which investigated federated learning in ASTARS-aided uplink networks to enable distributed model training while preserving user privacy and reducing communication overhead. These emerging research directions highlight the versatility of STAR-RIS technology in addressing diverse challenges in next-generation wireless networks.

### Motivation and contributions

Modern wireless communication paradigms require the integration of RIS and NOMA to meet the stringent QoS and throughput requirements of next-generation networks. Prior research has established that adaptive RIS-assisted NOMA configurations offer superior reliability and security for non-sensitive QoS users compared to static frameworks. Specifically, the authors in^20^ demonstrated that channel-aware adaptations yield significant reductions in outage events over Fisher-Snedecor-F fading channels. Similarly, the elevation-aware interference management framework introduced in^21^ highlighted the necessity of adaptive weighting factors to mitigate fluctuating interference inherent in integrated satellite-terrestrial mobility scenarios. These studies underscore a fundamental shift toward dynamic network architectures that can autonomously react to environmental variations.

A critical evolution in this domain is the transition from conventional reflecting-only RIS to simultaneously transmitting and reflecting RIS (STAR-RIS), which provides the 360-degree full-plane coverage essential for dense urban vehicular deployments. This architectural shift is particularly vital for vehicle-to-vehicle (V2V) and vehicle-to-everything (V2X) communications, where user mobility and blockage frequently create “dead zones”^22,23^. While previous investigations into STAR-RIS V2V systems under double-Rayleigh and Rician fading have provided foundational insights into high-SNR slopes and ergodic capacity, they often rely on fixed partitioning or energy-splitting protocols that do not fully exploit the instantaneous spatial and power-domain degrees of freedom.

The gain-adaptive feature further enhances spectral efficiency by dynamically adjusting to varying channel conditions and user requirements, enabling STAR-RIS to dynamically respond to changing channel conditions, optimizing beamforming, maximizing the efficiency of SIC decoding and enhancing spectral efficiency in complex environments^24^. Thus, addressing these issues through advanced optimization techniques is crucial for reducing interference, which are critical for vehicular communications and the successful deployment of these systems in next-generation wireless vehicular networks^25^. Throughout this paper, the term ’gain-adaptive’ refers to a dual mechanism that simultaneously responds to instantaneous channel gain conditions: dynamically reordering the SIC decoding sequence and reallocating the NOMA power coefficient based on users’ instantaneous CSI and target rate requirements, and fairness considerations.

While reference^11^ established the foundational outage and ergodic rate analysis for STAR-RIS-assisted NOMA under Rician fading, it assumes a fixed user ranking where the near/far designation and SIC decoding order remain static. This assumption is fundamentally incompatible with vehicular environments, where high mobility causes user channel gains to invert rapidly. Our work directly addresses this gap by introducing a dynamic gain-adaptive SIC ordering and power reallocation mechanism that responds to instantaneous CSI. Furthermore, we integrate a multi-antenna transmit antenna selection (TAS) technique at the BS – absent from reference^11^ – to exploit spatial diversity and counteract vehicular penetration losses. Finally, we introduce the Multiple Averaging Ergodic Sum Rate (MA-ESR), a novel metric that captures the joint statistical coupling between NOMA users in a STAR-RIS environment. Unlike the standard ergodic rate in^11^, MA-ESR provides a more accurate characterization of delay-tolerant vehicular network performance.

Despite these advances, a significant research gap remains: the majority of existing STAR-RIS NOMA literature^20–23^ assumes a fixed user ranking scenario, which is fundamentally incompatible with the rapidly changing channel conditions of vehicular networks. In such high-mobility environments, the “near” and “far” user designations can invert instantaneously, rendering static SIC orders inefficient. Addressing these limitations mandates a holistic, gain-adaptive framework designed to maintain optimal performance under stochastic channel variations. Our key contributions are summarized as follows:

- *Novel Dynamic Gain-Adaptive STAR-RIS NOMA (ASRN) Framework* We propose ASRN scheme that dynamically reorders SIC decoding and reallocates power based on instantaneous CSI. This ensures the system remains adaptable to the rapid topological changes inherent in vehicular networks.
- *Spatial diversity via transmit antenna selection (TAS) integration* We integrate a TAS technique at the multi-antenna BS to serve single-antenna vehicles. We analytically demonstrate that this configuration exploits spatial diversity to provide a reliability boost that scales with the number of antennas, effectively counteracting the penetration losses common in public transportation environments.
- *Closed-form outage and asymptotic analysis* We derive exact closed-form expressions for the system outage and asymptotic outage probability. We mathematically prove that the proposed ASRN scheme achieves a diversity order proportional to the number of antennas and a coding gain proportional to the number of STAR-RIS elements, providing a clear blueprint for system scaling.
- *Joint optimization via greedy algorithm* We introduce a computationally efficient greedy algorithm to solve the joint optimization problem of antenna selection and NOMA user pairing. This provides a practical balance between optimal performance and the low-latency processing requirements of real-time vehicular networks.
- *Ergodic Sum Rate (ESR) performance evaluation* To study the impact of ASRN in delay-tolerant network mode, we derive an analytical expression for the ESR performance. We provide a rigorous comparison against CSRN, ASRO, and CSRO schemes, revealing that ASRN achieves significantly higher average achievable data rates.
- *Introduction of the MA-ESR Metric* To better evaluate delay-tolerant vehicular networks, we define and derive the MA-ESR. This metric uniquely accounts for the joint variability and statistical coupling of NOMA users in a STAR-RIS environment, offering a more accurate performance measure than traditional ESR.
- *Extensive simulation and benchmarking* Through extensive Monte Carlo simulations, we validate that ASRN significantly outperforms four baseline schemes: dynamic OMA (ASRO), conventional STAR-RIS NOMA/OMA (CSRN/CSRO), and both Half-Duplex (HD) and Full-Duplex (FD) Decode-and-Forward (DF) relaying schemes, particularly in terms of outage performance, coding gain, and ESR performance in both delay-limited and delay-tolerant network modes.

### Organization and notations

The remainder of the paper is organized as follows. In Section System model, the system model is investigated, including the network structure, composite signal formulas and statistics. Section Outage performance analysis presents a detailed evaluation of the outage performance of the proposed and comparative schemes. Section Ergodic sum rate introduces ESR and MA-ESR as performance matrices to measure the average achievable data rate in the delay-tolerant network mode of operation. In Section Numerical results, the numerical results are provided. Then, some conclusions for the proposed scheme are investigated in Section Conclusion.

Notations: In this paper, vectors are represented by boldface letters. $\gamma \left( .,.\right)$, $\Gamma \left( .,.\right)$ and $\Gamma \left( .\right)$ represent the lower incomplete, the upper incomplete and the complete gamma functions, respectively. The cumulative distribution function (CDF), the complementary CDF and the probability density function (PDF) of a random variable *x* are given as ${F}_{X} \left( x\right)$, ${\bar{F}}_{X} \left( x\right)$ and ${f}_{X} \left( x\right)$, respectively. $\mathbb {E}$, $\mathbb {C}{^{m \times n}}$ and $\mathbb {P}$ are the statistical expectation, a complex-valued matrix of size $m \times n$, and the probability function, respectively. $I_0 \left( .\right)$ denotes the modified Bessel function of the first kind and rank zero. $\mathscr{C}\mathscr{N}\left( {0,\sigma } \right)$ is the normal distribution with zero mean and variance $\sigma$. Furthermore, Table 1 summarizes some important symbols and functions that are utilized in this paper with their corresponding definition. $$G_{p,q}^{m,n}\Big ( x \Big \vert \begin{matrix} a_1, \dots , a_n, a_{n+1}, \dots , a_p \\ b_1, \dots , b_m, b_{m+1}, \dots , b_q \end{matrix} \Big )$$ and $_2F_1 \left( .\right)$ represent the Meijer-G function^26^, Eq. (9.30) and the Gauss hypergeometric function^26^, Eq. (9.14.2), respectively.

**Table 1:** Some important symbols and functions (S&F) used in this paper.

S&F	Description
$\rho$	The transmit SNR
${\mathbb {U}}_r$	Reflection user with respect to STAR-RIS
${\mathbb {U}}_t$	Transmission user with respect to STAR-RIS
$d_{b}$ and $d_{{\mu }}$	The distances from BS to STAR-RIS and STAR-RIS to ${\mathbb {U}}_\mu$, $\mu \in \left\{ {t,r} \right\}$, respectively.
$\boldsymbol{\phi } _n^r$	The reflection phase shifts of the *n*^th^ element of STAR-RIS to ${\mathbb {U}}_r$
$\boldsymbol{\phi } _n^t$	The transmission phase shifts of the *n*^th^ element of STAR-RIS to ${\mathbb {U}}_t$
$\lambda _r$	The energy-splitting reflection coupling coefficient of STAR-RIS
$\lambda _t$	The energy-splitting transmission coupling coefficient of STAR-RIS
${\mathbb {U}}_{\textbf{n}}$	Near user with respect to STAR-RIS where $\textbf{n} \in \left\{ {t,r} \right\}$
${\mathbb {U}}_{\textbf{f}}$	Far user with respect to STAR-RIS where $\textbf{f} \in \left\{ {\left\{ {t,r} \right\} -\left\{ {\textbf{n}} \right\} } \right\}$
${{x}_{\textbf{n}}}$ and ${{x}_{\textbf{f}}}$	The symbols of ${\mathbb {U}}_{\textbf{n}}$ and ${\mathbb {U}}_{\textbf{f}}$, respectively
$\alpha$ and $1-\alpha$	The power allocation coefficients of ${{x}_{\textbf{n}}}$ and ${{x}_{\textbf{f}}}$, respectively
${{\boldsymbol{\Phi }} _\textbf{n}}$	STAR-RIS phase shifting matrix to ${\mathbb {U}}_{\textbf{n}}$ where $\textbf{n} \in \left\{ {t,r} \right\}$
${{\boldsymbol{\Phi }} _\textbf{f}}$	STAR-RIS phase shifting matrix to ${\mathbb {U}}_{\textbf{f}}$ where $\textbf{f} \in \left\{ {\left\{ {t,r} \right\} -\left\{ {\textbf{n}} \right\} } \right\}$
$g_{\textbf{n}}$	The cascaded channel gain coefficient from BS to ${\mathbb {U}}_{\textbf{n}}$
$g_{\textbf{f}}$	The cascaded channel gain coefficient from BS to ${\mathbb {U}}_{\textbf{f}}$
$\zeta$	The path loss exponent
$\kappa$	The Rician factor
${\eta }_{\textbf{f}}\left( \alpha ,x\right)$	$= \frac{x}{\rho \left( 1-\alpha -\alpha x\right) }$; an arbitrary function describes a lower bound on $g_{\textbf{f}}$ for a specific target value *x* in case of NOMA network
${\psi }_{\textbf{n}}\left( \alpha ,x\right)$	$=\frac{x}{\rho \alpha }$ ; an arbitrary function describes a lower bound on $g_{\textbf{n}}$ for a specific target value *x* in case of NOMA network
${\eta }_{\textbf{f}}^{\star }\left( \alpha ,x\right)$	$= \frac{x}{\rho \left( 1-\alpha \right) }$ ; an arbitrary function describes a lower bound on $g_{\textbf{f}}$ for a specific target value *x* in case of OMA network
${\psi }_{\textbf{n}}^{\star }\left( \alpha ,x\right)$	$=\frac{x}{\rho \alpha }$ ; an arbitrary function describes a lower bound on $g_{\textbf{n}}$ for a specific target value *x* in case of OMA network

## System model

**Fig. 1:**
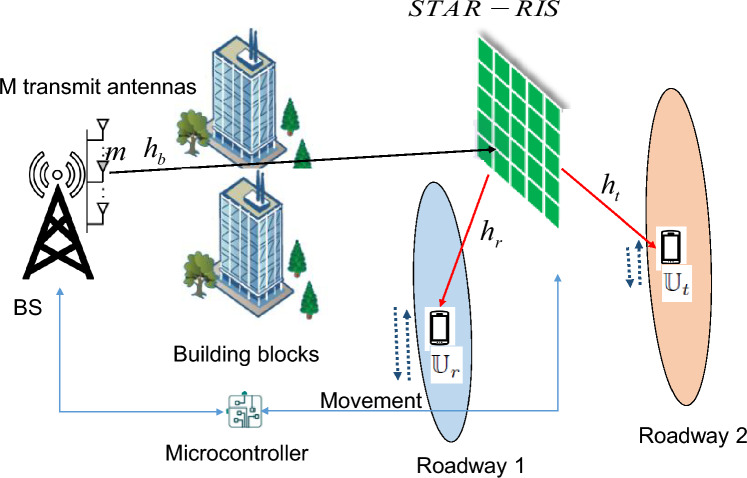
The adaptive STAR-RIS assisted vehicular NOMA system with a TAS technique.

Consider a downlink STAR-RIS NOMA network that consists of a base station (BS) which communicates with two vehicular NOMA users^1^, i.e., termed as $\mathbb {U}_r$ and $\mathbb {U}_t$, only via the assistance of a STAR-RIS equipment as shown in Fig. 1. There is no direct link between the BS and the two users due to heavy blockage. Thus, the BS needs the assistance of STAR-RIS to communicate with the users.

The BS is equipped with *M* antennas to transmit signals to the two users that are equipped with only one antenna each, while the STAR-RIS equipment contains *N* reflecting and refracting meta-elements that are able to reflect and refract the incident signal to $\mathbb {U}_r$ and $\mathbb {U}_t$, respectively. This topology can be founded in a cellular system with multiple roadway services in which the BS selects the optimal antenna to communicate with distant vehicular users of different roadways located in the vicinity of the reflection and/or transmission regions of a mounted STAR-RIS behind different types of blockage.

The practical necessity of STAR-RIS over conventional reflecting-only RIS is especially pronounced in dense urban vehicular environments. Conventional RIS is restricted by a ’half-plane’ coverage constraint, which creates significant geographical blind spots in roadway deployments. These limitations are critical when users are distributed on both sides of a surface, such as an RIS mounted on a glass-walled building or a median partition. By enabling simultaneous reflection and transmission, STAR-RIS achieves full-plane (360-degree) coverage. This capability allows the BS to pair a ’reflected’ user with a ’transmitted’ user within a single resource block. Consequently, the proposed scheme overcomes the geometrical limitations of traditional surfaces to ensure robust connectivity in complex urban topologies.

Let the BS selects an antenna *m* out of multiple *M* antennas (ordered as $\left\{ 1, \dots , m, \dots , M\right\}$ ) by employing a particular transmit antenna selection technique (TAS) to provide a diversity gain and reduce implementation complexity. The channel coefficients from the selected antenna of the BS to STAR-RIS and STAR-RIS to user $\textrm{U}_{\mu }$ are denoted as ${\mathbf{{h}}_{b}} \in \mathbb {C}{^{N \times 1}}$ and ${{\mathbf{{h}}_{\mu } }} \in \mathbb {C}{^{N \times 1}}$ with $\mu \in \left\{ {t,r} \right\}$, respectively. Then, ${\mathbf{{h}}_{b}}$ and ${{\mathbf{{h}}_{\mu } }}$ are considered to be independent and identically distributed (iid) channel coefficients. All wireless communication channels are considered to experience Rician fading as a practical channel fading approach. Assuming that the STAR-RIS operates in energy-splitting (ES) mode with independent transmission and reflection phase shifting of STAR-RIS (as in^27^), we denote ${{\boldsymbol{\Phi }} _r} = \sqrt{ {\lambda _r}} \mathrm{{diag}}\left( {{e^{j\boldsymbol{\phi }_1^r}},...,{e^{j\boldsymbol{\phi } _n^r}},...,{e^{j\boldsymbol{\phi } _N^r}}} \right)$ and ${{\boldsymbol{\Phi }} _t} = \sqrt{ {\lambda _t}} \mathrm{{diag}}\left( {{e^{j\boldsymbol{\phi } _1^t}},...,{e^{j\boldsymbol{\phi } _n^t}},...,{e^{j\boldsymbol{\phi } _N^t}}} \right)$ the reflection and transmission phase shifting matrices, ${\boldsymbol{\phi } _n^r}$, ${\boldsymbol{\phi } _n^t} \in \left[ {0,2\pi } \right]$ represent the reflection and transmission phase shifts of the *n*^th^ element of STAR-RIS, respectively, where ${\lambda _r}$ and ${\lambda _t}$ are the reflection and transmission coupling coefficients of STAR-RIS, respectively, with ${\lambda _r} + {\lambda _t} = 1$.

Let ${{g}_{\mu }}={{\left| {\mathbf{{h}}_{\mu }^H{{\boldsymbol{\Phi }} _\mu }{\mathbf{{h}}_{b}}} \right| }^2}$ denote the cascaded channel gain coefficients from the selected antenna of the BS to ${\mathbb {U}}_\mu$ with $\mu \in \left\{ {t,r} \right\}$, where ${\mathbf{{h}}_{b} } = \sqrt{d_{b} ^{ - \zeta }} {\left[ {h_{b}^1, \cdots ,h_{b} ^n, \cdots ,h_{b} ^N} \right] ^H}$ and ${\mathbf{{h}}_{\mu } } = \sqrt{d_{\mu } ^{ - \zeta }} {\left[ {h_{\mu } ^1, \cdots ,h_{\mu } ^n, \cdots ,h_{\mu } ^N} \right] ^H}$, $h_{b}^n = {\sqrt{\frac{\kappa }{{\kappa + 1}}} + \sqrt{\frac{1}{{\kappa + 1}}} \tilde{h}_{b}^n}$, $\tilde{h}_{b }^n \sim \mathscr{C}\mathscr{N}\left( {0,1} \right)$ and $h_{\mu } ^n = {\sqrt{\frac{\kappa }{{\kappa + 1}}} + \sqrt{\frac{1}{{\kappa + 1}}} \tilde{h}_{{\mu }} ^n}$, $\tilde{h}_{\mu }^n \sim \mathscr{C}\mathscr{N}\left( {0,1} \right)$, respectively, where $\zeta$ denotes the path loss exponent, $\kappa$ is the Rician factor, $d_{b}$ and $d_{{\mu }}$ are the distances from the selected antenna of the BS to STAR-RIS and STAR-RIS to ${\mathbb {U}}_\mu$, respectively.

According to the NOMA principle, one of the two users can represent a far user denoted by ${\mathbb {U}}_{\textbf{f}}$, $\textbf{f} \in \left\{ {t,r} \right\}$, and the other represents a near user denoted by ${\mathbb {U}}_{\textbf{n}}$, $\textbf{n} \in \left\{ {\left\{ {t,r} \right\} -\left\{ {\textbf{f}} \right\} } \right\}$. The BS superposes the signal ${x}_{BS}={{x}_{\textbf{n}}}\sqrt{P\alpha }+{{x}_{\textbf{f}}}\sqrt{P \left( 1-\alpha \right) }$ where *P* is the BS transmit power, $\alpha$ is the power allocation coefficient, ${{x}_{\textbf{n}}}$ and ${{x}_{\textbf{f}}}$ are the symbols of ${\mathbb {U}}_{\textbf{n}}$ and ${\mathbb {U}}_{\textbf{f}}$, respectively, where $\mathbb {E}\left\{ {{\left| {{x}_{\textbf{n}}} \right| }^{2}} \right\} =\mathbb {E}\left\{ {{\left| {{x}_{\textbf{f}}} \right| }^{2}} \right\} =1$. The received signal at ${\mathbb {U}}_{\textbf{f}}$ and ${\mathbb {U}}_{\textbf{n}}$ can be respectively expressed as

1 $$\begin{aligned} {y_\textbf{f}} = {\mathbf{{h}}_{\textbf{f}}^H{{\boldsymbol{\Phi }} _\textbf{f}}{\mathbf{{h}}_{b}}} x_{BS} + {{\sigma }_\textbf{f}}, \end{aligned}$$

2 $$\begin{aligned} {y_\textbf{n}} = {\mathbf{{h}}_{\textbf{n}}^H{{\boldsymbol{\Phi }} _\textbf{n}}{\mathbf{{h}}_{b}}} x_{BS} + {{\sigma }_\textbf{n}}, \end{aligned}$$

where ${\sigma }_\textbf{f}, {\sigma }_\textbf{n} \sim \mathscr{C}\mathscr{N} \left( {0,\sigma _0} \right)$ are the thermal noise at ${\mathbb {U}}_{\textbf{f}}$ and ${\mathbb {U}}_{\textbf{n}}$ with a variance of $\sigma _0$, respectively. Hence, ${\mathbb {U}}_{\textbf{f}}$ decodes its symbol ${{x}_{\textbf{f}}}$ directly by considering ${{x}_{\textbf{n}}}$ as a noise, whereas the ${\mathbb {U}}_{\textbf{n}}$ can successfully extract ${{x}_{\textbf{f}}}$ by treating its symbol ${{x}_{\textbf{n}}}$ as a noise. Then, it decodes ${{x}_{\textbf{n}}}$ by performing SIC technique. Let $g_{\textbf{f}}={{\left| {\mathbf{{h}}_{\textbf{f}}^H{\boldsymbol{\Phi }} _\textbf{f}{\mathbf{{h}}_{b}}} \right| }^2}$ and $g_{\textbf{n}}={{\left| {\mathbf{{h}}_{\textbf{n}}^H{\boldsymbol{\Phi }} _\textbf{n}{\mathbf{{h}}_{b}}} \right| }^2}$, the received SNR at ${\mathbb {U}}_{\textbf{f}}$ is expressed as

3 $$\begin{aligned} \gamma _{\textbf{f}}=\frac{\rho \left( 1-\alpha \right) g_{\textbf{f}}}{\rho \alpha g_{\textbf{f}}+1} \end{aligned}$$

where $\rho = \frac{P}{{{\sigma }_{0}}}$ is the transmit SNR. The received SNRs at ${\mathbb {U}}_{\textbf{n}}$ to detect ${{x}_{\textbf{f}}}$ and extract ${{x}_{\textbf{n}}}$ can be expressed respectively as

4 $$\begin{aligned} \gamma _{\textbf{n} \rightarrow \textbf{f}}=\frac{\rho \left( 1-\alpha \right) g_{\textbf{n}}}{\rho \alpha g_{\textbf{n}}+1} \end{aligned}$$

5 $$\begin{aligned} \gamma _{\textbf{n}}=\rho \alpha g_{\textbf{n}}. \end{aligned}$$

To maximize the received signal power at both users, a coherent phase shifting scheme is employed at the STAR-RIS. Specifically, the phase shifts ${\boldsymbol{\phi } _n^r}$ and ${\boldsymbol{\phi } _n^t}$, $n \in \left\{ 1,2,\dots ,N \right\}$, are configured to align (compensate) the channels’ phases of ${\mathbb {U}}_r$ and ${\mathbb {U}}_t$, respectively. This is achieved by setting ${\boldsymbol{\phi } _n^r} = -\arg \left( h_r^n h_b^n\right)$ and ${\boldsymbol{\phi } _n^t} = -\arg \left( h_t^n h_b^n\right)$, with $\cos {\left( {\boldsymbol{\phi } _n^t}-{\boldsymbol{\phi } _n^r}\right) }=0$, where $\arg \left( \cdot \right)$ denotes the phase angle. By compensating the phase shifts of the cascaded channels, all *N* elements contribute constructively to the received signal, thereby achieving coherent combining and maximizing the channel gain.

Under this coherent phase alignment,, ${{g}_{\mu }}$ can be expressed as ${{g}_{\mu }}=d_{b} ^{ - \zeta }d_{\mu } ^{ - \zeta }\lambda _{\mu }Z_{{\mu }}$, where $Z_{{\mu }}=\left| {\sum \nolimits _{n = 1}^N {\left| {h_{\mu }^n h_{b}^n} \right| } } \right| ^2$ with $\mu \in \left\{ {{\textbf{n}},{\textbf{f}}} \right\}$. Based on full CSI at BS^12^, a dynamic ranking is performed to determine whether ${\mathbb {U}}_r$ or ${\mathbb {U}}_t$ can represent ${\mathbb {U}}_{\textbf{f}}$ or ${\mathbb {U}}_{\textbf{n}}$ which is denoted by $\left\{ \left( \textbf{n},\textbf{f} \right) \right\} \in \left\{ {\left( t,r \right) ,\left( r,t \right) } \right\}$ based on the following condition,

6 $$\begin{aligned} \left( \textbf{n},\textbf{f} \right) =\quad \!\!\!{\left\{ \begin{array}{ll} \left( t,r \right) ,& \quad g_t > g_r \\ \left( r,t \right) ,& \quad g_t < g_r . \end{array}\right. } \end{aligned}$$

In the context of STAR-RIS assisted vehicular NOMA with channel gain adaptation, focusing on channel-aware design, adaptive mechanisms, and vehicular dynamics, the Gamma approximation can be used to model the characteristics of the composite fading channels for mathematical tractability. It can be derived effectively via moment matching by matching the first and second moments (mean and variance) of the true distribution to a Gamma distribution^28^. According to^11^ for large *N* meta-elements, the Central Limit Theorem (CLT) is often used alongside the Gamma approximation to model the cascaded channels. The CDF and PDF of $Z_{\mu }$, $\mu \in \left\{ {\textbf{n}},{\textbf{f}}\right\}$, $\textbf{n} \in \left\{ {t,r} \right\}$, $\textbf{f} \in \left\{ {\left\{ {t,r} \right\} -\left\{ {\textbf{n}} \right\} } \right\}$, can be approximated respectively by

7 $$\begin{aligned} {F_{Z_{\mu }}}\left( x \right) \approx \frac{\gamma \left( {{E_{\mu } },\frac{{\sqrt{x} }}{{{V_{\mu } }}}} \right) }{\Gamma {\left( {{E_{\mu } }} \right) }}\overset{(\ddagger )}{\approx }\ 1-\frac{\Gamma \left( {{E_{\mu } },\frac{{\sqrt{x} }}{{{V_{\mu } }}}} \right) }{\Gamma {\left( {{E_{\mu } }} \right) }}, \end{aligned}$$

8 $$\begin{aligned} {f_{Z_{\mu }}}\left( x \right) \approx \frac{{{x^{\frac{{E_\mu }}{2} - 1}}}}{{2 {\left( {V_\mu } \right) }^{E_\mu }\Gamma \left( {{{E_\mu }}} \right) }}{e^{ - \frac{{\sqrt{x} }}{{V_\mu }}}}, \end{aligned}$$

where ${E_{\mu } } = \frac{{N{\mathscr {E}^2}}}{\mathscr {V}}$, ${V_{\mu } } = \frac{{\mathscr {V}}}{{\mathscr {E}}}$, $\mathscr {V} = {1 - \frac{{{\pi ^2}}}{{16{{\left( {\kappa + 1} \right) }^2}}}{{\left[ {{L_{\frac{1}{2}}}\left( { - \kappa } \right) } \right] }^4}}$, $\mathscr {E} = \frac{{\pi }}{{4\left( {\kappa + 1} \right) }}{\left[ {{L_{\frac{1}{2}}}\left( { - \kappa } \right) } \right] ^2}$,${L_{\frac{1}{2}}}\left( x \right) = \sqrt{e^x} \big [ {\left( {1 - x } \right) {I_0}\left( { - \frac{x }{2}} \right) - x {I_1}\left( { - \frac{x }{2}} \right) } \big ]$ is the Laguerre polynomial, $I_0 \left( .\right)$ is the modified Bessel function of the first kind and rank zero, $\gamma \left( {a,x} \right) = \int _0^x {{y^{a - 1}}{e^{ - y}}dy}$ denotes the lower incomplete Gamma function^26^, Eq. (8.350.1), $\Gamma \left( {a,x} \right)$ denotes the upper incomplete Gamma function^26^, Eq. (8.350.2), $\Gamma \left( \cdot \right)$ is the gamma function^26^, Eq. (8.310.1) and $(\ddagger )$ follows by using the relation $\Gamma \left( {a,x} \right) +\gamma \left( {a,x} \right) =\Gamma \left( {a} \right)$^26^, Eq. (8.356.3). Furthermore, we define the functions ${\eta }_{\textbf{f}}\left( \alpha ,x\right) = \frac{x}{\rho \left( 1-\alpha -\alpha x\right) }$ where $1-\alpha -\alpha x > 0 \Rightarrow x < \frac{1-\alpha }{\alpha }$, ${\psi }_{\textbf{n}}\left( \alpha ,x\right) =\frac{x}{\rho \alpha }$, ${\eta }_{\textbf{f}}^{\star }\left( \alpha ,x\right) = \frac{x}{\rho \left( 1-\alpha \right) }$ and ${\psi }_{\textbf{n}}^{\star }\left( \alpha ,x\right) =\frac{x}{\rho \alpha }$. Then, the first derivatives with respect to *x* are given as $\frac{\partial {\eta }_{\textbf{f}}\left( \alpha ,x\right) }{\partial x}={\eta }^{'}_{\textbf{f}}\left( \alpha ,x\right) = \frac{1-\alpha }{\left[ \rho \left( 1-\alpha -\alpha x\right) \right] ^2}$, ${\psi }^{'}_{\textbf{n}}\left( \alpha ,x\right) =\frac{1}{\rho \alpha }$, ${\eta }^{\star '}_{\textbf{f}}\left( \alpha ,x\right) = \frac{1}{ \rho \left( 1-\alpha \right) }$ and ${\psi }^{\star '}_{\textbf{n}}\left( \alpha ,x\right) =\frac{1}{\rho \alpha }$, respectively.

In this study, perfect CSI is assumed at the BS to facilitate the derivation of fundamental performance bounds. However, in practical vehicular scenarios, high mobility leads to rapid channel fluctuations and significant Doppler spreads, which complicate the estimation process. Imperfect CSI can introduce residual interference during the SIC process and increase the signaling overhead required for frequent channel feedback. While these factors may degrade the absolute performance, the current analysis serves as a critical theoretical upper bound. Furthermore, it provides the necessary mathematical framework to which estimation error models can be applied in future extensions of this work. While vehicle speed and Doppler shift are not explicitly represented as variables, the proposed ASRN framework directly addresses the high channel volatility inherent in vehicular environments. In high-mobility V2X scenarios, the relative channel gains of vehicular users fluctuate rapidly; the proposed ASRN scheme responds to this by performing instantaneous CSI-based SIC reordering and power reallocation to maintain connectivity at every channel realization.

## Outage performance analysis

In this section, we derive analytical expressions for the system outage probability, asymptotic outage probability and diversity order.

### NOMA

Let ${\eta }_{\textbf{f}}\left( \alpha \right) ={\eta }_{\textbf{f}}\left( \alpha ,{{\bar{\gamma }} _{\textbf{f}}}\right) =\frac{{\bar{\gamma }} _{\textbf{f}}}{\rho \left( 1-\alpha -\alpha {\bar{\gamma }} _{\textbf{f}} \right) }$, ${\psi }_{\textbf{n}}\left( \alpha \right) ={\psi }_{\textbf{n}}\left( \alpha ,{{\bar{\gamma }} _{\textbf{n}}}\right) =\frac{{\bar{\gamma }} _{\textbf{n}}}{\rho \alpha }$, ${\bar{\gamma }}_\varLambda =2^{R_{\varLambda }}-1$, $\varLambda \in \left\{ {\textbf{n},\textbf{f}} \right\}$ and $R_{\varLambda }$ are target rates. Since the system outage occurs if ${\mathbb {U}}_{\textbf{f}}$ or ${\mathbb {U}}_{\textbf{n}}$ fails to decode its own symbol, the detection probability^7^ implies that the events $g _{\textbf{f}}\ge {\eta }_{\textbf{f}}\left( \alpha \right)$, $g _{\textbf{n}} \ge {\eta }_{\textbf{f}}\left( \alpha \right)$ and $g _{\textbf{n}}\ge {\psi }_{\textbf{n}}\left( \alpha \right)$ should be jointly satisfied. Considering that the *m*^th^ antenna is the selected antenna at the BS, the system outage probability can be expressed as

9 $$\begin{aligned} \begin{aligned} P_{m}&=1- \mathbb {P}\left( {g _{\textbf{f}}\ge {\eta }_{\textbf{f}}\left( \alpha \right) ,g _{\textbf{n}}\ge \max \left( {\eta }_{\textbf{f}}\left( \alpha \right) ,{\psi }_{\textbf{n}}\left( \alpha \right) \right) } \right) \\ &\overset{(\dagger )}{\mathop {=}}\,1- \mathbb {P}\left( {g _{\textbf{f}}\ge {\eta }_{\textbf{f}}\left( \alpha \right) ,g_{\textbf{n}}\ge {\psi }_{\textbf{n}}\left( \alpha \right) } \right) =\mathbb {P}\left( {\min \left( \frac{g _{\textbf{f}}}{{\eta }_{\textbf{f}}\left( \alpha \right) },\frac{g_{\textbf{n}}}{{\psi }_{\textbf{n}}\left( \alpha \right) }\right) } < 1 \right) , \end{aligned} \end{aligned}$$

where $(\dagger )$ follows as $g _{\textbf{n}}\ge {\eta }_{\textbf{f}}\left( \alpha \right)$ is always satisfied whenever $g _{\textbf{f}}\ge {\eta }_{\textbf{f}}\left( \alpha \right)$ is satisfied. Furthermore, the BS leverages spatial diversity (e.g., the selection from multiple antennas) for better signal quality and fairness, using techniques like TAS to reduce hardware cost (e.g., fewer RF chains) and complexity. The optimal *m*^th^ antenna (denoted by ${m^*}$) is selected according to the following condition

10 $$\begin{aligned} {m^*}=\underset{m \in \left\{ 1,2,.......,M \right\} }{\mathop {\arg \max }}\,{\min \left( \frac{g _{\textbf{f}}}{{\eta }_{\textbf{f}}\left( \alpha \right) },\frac{g _{\textbf{n}}}{{\psi }_{\textbf{n}}\left( \alpha \right) }\right) }.\end{aligned}$$

#### Theorem 2

The system outage probability of the proposed ASRN scheme is expressed as (11) at the top of the page where $\eta _{\textbf{f}}={\eta }_{\textbf{f}}\left( \alpha \right)$, $\psi _{\textbf{n}}={\psi }_{\textbf{n}}\left( \alpha \right)$ for an arbitrary fixed $\alpha < \frac{1}{ \left( 1+ \max \left( {\bar{\gamma }}_{\textbf{f}},{\bar{\gamma }}_{\textbf{n}} \right) \right) }$, $E_{{\mu }}=E$, $V_{{\mu }}=V$ and ${\bar{F}}_{X} \left( x\right) =1-{F}_{X} \left( x\right)$ for a specific random variable *X*.

11 $$\begin{aligned}&P_o=\bigg [1- \sum \limits _{p = 0}^\infty {\frac{\left( -1\right) ^p }{p! \left( E+p\right) \left( \Gamma {\left( {E} \right) }\right) ^2} \left( \left( {\sqrt{\frac{U_r}{U_t}} }\right) ^{E+p}\Gamma \left( {2E+p,\frac{\sqrt{\psi _r} }{\sqrt{U_r}V }} \right) +\left( {\sqrt{\frac{U_t}{U_r}} }\right) ^{E+p}\Gamma \left( {2E+p,\frac{\sqrt{\psi _t} }{\sqrt{U_t}V }} \right) \right) } \nonumber \\&\quad +\frac{\Gamma \left( {E,\frac{{\sqrt{\psi }_r }}{{\sqrt{U_r} V }}} \right) +\Gamma \left( {E,\frac{{\sqrt{\psi }_t }}{{\sqrt{U_t} V }}} \right) }{\Gamma {\left( {E} \right) }}-\frac{1}{\left( \Gamma {\left( {E} \right) }\right) ^2} \left( \Gamma \left( {E,\frac{{\sqrt{\eta }_t }}{{\sqrt{U_t} V }}} \right) \Gamma \left( {E,\frac{{\sqrt{\psi }_r }}{{\sqrt{U_r} V }}} \right) + \Gamma \left( {E,\frac{{\sqrt{\eta }_r }}{{\sqrt{U_r} V }}} \right) \Gamma \left( {E,\frac{{\sqrt{\psi }_t }}{{\sqrt{U_t} V }}} \right) \right) \bigg ]^M. \end{aligned}$$

#### Proof

Based on (10), the system outage probability is as

12 $$\begin{aligned} \begin{aligned} P_o = \underset{m }{\mathop {\max }}\,P_{m}&=\left( 1- \mathbb {P}\left( {g _{\textbf{f}}\ge {\eta }_{\textbf{f}}\left( \alpha \right) ,g_{\textbf{n}}\ge {\psi }_{\textbf{n}}\left( \alpha \right) } \right) \right) ^M \\ &=\bigg [1-\big [\underbrace{\mathbb {P}\left( {g_t}\ge \eta _t,{g_r}>{g_t},{g_r}\ge \psi _r \right) }_{\mathbb {P}_1}+\underbrace{\mathbb {P}\left( {g_r}\ge \eta _r,{g_t}>{g_r},{g_t}\ge \psi _t \right) }_{\mathbb {P}_2}\big ]\bigg ]^M. \end{aligned} \end{aligned}$$

As ${{g}_{\mu }}={U_{\mu }}Z_{{\mu }}$, where ${U_{\mu }}=d_{b} ^{ - \zeta }d_{\mu } ^{ - \zeta }\lambda _{\mu }$, the CDF and PDF of ${{g}_{\mu }}$ are given respectively by utilizing (7) and (8) as $F_{g_{\mu }}\left( x\right) =F_{Z_{\mu }}\left( x/U_{\mu }\right)$ and $f_{g_{\mu }}\left( x\right) =1/U_{\mu } f_{Z_{\mu }}\left( x/U_{\mu }\right)$. Hence, $\mathbb {P}_1$ is evaluated as follows

13 $$\begin{aligned} \mathbb {P}_1={{\bar{F}}_{{Z_t}}}\left( \eta _t/U_t \right) \bar{F}_{Z_{r}}\left( \psi _{r}/U_{r} \right) -\underbrace{ \int \limits _{\psi _{r} }^{\infty }{\frac{1}{U_{r}} \bar{F}_{Z_t}\left( x/U_t \right) f_{Z_{r}} \left( x/U_{r} \right) dx}}_{\mathbb {I}_1}, \end{aligned}$$

substitute (7) and (8) in $\mathbb {I}_1$, we can get

14 $$\begin{aligned} \mathbb {I}_1=\frac{{U_{r}^{-\frac{E_{r} }{2}}}}{{2 {\left( {V_{r}} \right) }^{E_{r} }{\Gamma \left( {{{E_{t} }}} \right) }{\Gamma \left( {{{E_{r} }}} \right) }}} \times \underbrace{\int \limits _{\psi _{r} }^{\infty }{{{{x^{\frac{{E_{r} }}{2} - 1}}}{e^{ -\frac{{\sqrt{x} }}{{{\sqrt{U_{r}} V_{r} }}}}}\Gamma \left( {{E_{t} },\frac{{\sqrt{x} }}{{{\sqrt{U_{t}} V_{t} }}}} \right) }dx}}_{\mathbb {I}_2}. \end{aligned}$$

To the best of our knowledge, solving $\mathbb {I}_2$ is a challenging task. By virtue of the series representation $\Gamma \left( c,y \right) =\Gamma \left( c \right) -\sum \limits _{p = 0}^\infty {\frac{\left( -1\right) ^p}{p! \left( c+p \right) } \left( y \right) ^{c+p}}$^26^, Eq. (8.354.2), $\mathbb {I}_2$ can be derived using^26^, Eq. (8.381.9) with further algebraic manipulations as

15 $$\begin{aligned} \mathbb {I}_2=2 \left( {\sqrt{U_{r}} V_{r}}\right) ^{ E_{r} }\Gamma \left( E_t\right) \Gamma \left( {E_{r},\frac{\sqrt{\psi _{r}} }{\sqrt{U_{r}}V_{r}} } \right) - \sum \limits _{p = 0}^\infty {\frac{2\left( -1\right) ^p \left( {\sqrt{U_{r}} V_{r}}\right) ^{ E_{r}+E_{t}+p }}{p! \left( E_{t}+p\right) \left( {\sqrt{U_{t}} V_{t}}\right) ^{E_{t}+p}}} \times {\Gamma \left( {E_{r}+E_{t}+p,\frac{\sqrt{\psi _{r}} }{\sqrt{U_{r}}V_{r}} } \right) }. \end{aligned}$$

For $E_{{t}}=E_{r}=E$ and $V_{{t}}=V_{r}=V$ while $U_{{t}} \ne U_{r}$, $\mathbb {P}_2$ can be evaluated in a similar way as $\mathbb {P}_1$. By gathering the terms of equal powers, (11) can be obtained. $\square$

#### Remark 1

The infinite summation in (11) is derived from the series representation of the upper incomplete gamma function $\Gamma \left( c,y \right) =\Gamma \left( c \right) -\sum \limits _{p = 0}^\infty {\frac{\left( -1\right) ^p}{p! \left( c+p \right) } \left( y \right) ^{c+p}}$^26^, Eq. (8.354.2). This series converges absolutely for all $y > 0$ as it satisfies the conditions of a Leibniz alternating series. Specifically, the ratio test yields $\lim _{p \rightarrow \infty } \left| \frac{a_{p+1}}{a_p} \right| = \lim _{p \rightarrow \infty } \frac{y}{E+p+1} = 0 < 1$, confirming absolute convergence. Specifically, the truncation error after $p_{max}$ terms is bounded by the magnitude of the first neglected term, expressed as $err=\frac{y^{c+p_{max} +1}}{\left( p_{max} +1\right) !\left( c+p_{max} +1\right) }$. Given the rapid growth of the factorial term $\{\left( p_{max} +1\right) !\}$, numerical convergence to an error tolerance of $10^{-6}$ can be typically achieved by $p_{max} \approx 5-10$ terms for the parameter ranges considered in this work, as the factorial growth in the denominator ensures rapid decay of successive terms.

To gain further insights on the system outage performance, we study the impact of increasing the transmit SNR. As $y\rightarrow 0$, $\gamma \left( c,y \right) \rightarrow \frac{1}{c} y^c$. Then, we have the following corollary.

#### Corollary 1

At high transmit SNR, i.e., $\rho \rightarrow \infty$, the system asymptotic outage probability of the proposed ASRN scheme can be approximated as,

16 $$\begin{aligned} P_{o}^{Asy.}\approx \bigg [1- \frac{\Gamma \left( 2E \right) \left( {{\left( \sqrt{\frac{{{U}_{r}}}{{{U}_{t}}}} \right) }^{E}}+{{\left( \sqrt{\frac{{{U}_{t}}}{{{U}_{r}}}} \right) }^{E}}\right) }{E \Gamma {{\left( E \right) }^{2}}} + \frac{{{\left( \frac{{{\sqrt{\eta }}_{t}}}{\sqrt{{{U}_{t}}}V} \right) }^{E}}+{{\left( \frac{{{\sqrt{\eta }}_{r}}}{\sqrt{{{U}_{r}}}V} \right) }^{E}}}{E \Gamma \left( E \right) } +\frac{{{\left( \frac{{{\psi }_{r}}}{\sqrt{{{U}_{t}}{{U}_{r}}}{{V}^{2}}} \right) }^{E}}+{{\left( \frac{{{\psi }_{t}}}{\sqrt{{{U}_{t}}{{U}_{r}}}{{V}^{2}}} \right) }^{E}}}{2{{E}^{2}} \Gamma {{\left( E \right) }^{2}}} + \mathscr {O}\left( \frac{1}{\rho }\right) \bigg ]^M, \end{aligned}$$

where $\mathscr {O}\left( \frac{1}{\rho }\right)$ denotes higher power functions of $\frac{1}{\rho }$.

#### Proof

By using $\Gamma \left( c,y \right) \rightarrow \gamma \left( c \right) -\frac{1}{c} y^c$ as $y \rightarrow 0$ and neglecting higher power ranks than first rank power terms in (11), we can obtain (16). $\square$

It is notable that the system outage performance can be characterized by the rate at which the outage probability decreases as $\rho \rightarrow \infty$. The diversity order is defined as

17 $$\begin{aligned} \textbf{D} =-\underset{\rho \rightarrow \infty }{\mathop {\lim }}\,\frac{\log \left( P_{o}^{Asy.}\left( \rho \right) \right) }{\log \left( \rho \right) }.\end{aligned}$$

#### Remark 2

The outage behavior of ASRN scheme can achieve a diversity gain scaled with *M*.

#### Remark 3

In (16), the outage performance contains sum of terms that have dominant terms scaled with $\rho$ as $\left( \sqrt{\rho }\right) ^{-E}=\left( \sqrt{\rho }\right) ^{-\frac{{N{\mathbb {E}^2}}}{\mathbb {V}}}$. Thus, the system can achieve a coding gain increases with the number of STAR-RIS elements, i.e., *N*.

To optimize system outage performance, we perform a dynamic gain adaptation for the system considered in^29^. The power allocation scheme was designed to ensure that the near (SIC) user’s QoS is prioritized where $\alpha$ can be derived based on the users’ target rates as

18 $$\begin{aligned} \alpha =\frac{{\bar{\gamma }} _{\textbf{n}}}{{\bar{\gamma }} _{\textbf{f}}{\bar{\gamma }} _{\textbf{n}}+{\bar{\gamma }} _{\textbf{f}}+ {\bar{\gamma }} _{\textbf{n}}}.\end{aligned}$$

This adaptive power allocation, combined with the dynamic SIC ordering in (6), constitutes the gain-adaptive mechanism of the proposed ASRN scheme. Since $\alpha _{min}$ is a function of the target rates, the distribution of power adapts to specific user QoS requirements, ensuring a fair allocation that minimizes the joint outage probability.

#### Lemma 1

$\alpha _{min}=\frac{{\bar{\gamma }} _{\textbf{n}}}{{\bar{\gamma }} _{\textbf{f}}{\bar{\gamma }} _{\textbf{n}}+{\bar{\gamma }} _{\textbf{f}}+ {\bar{\gamma }} _{\textbf{n}}}$ is the optimal $\alpha$ for minimum outage at ${\mathbb {U}}_{\textbf{n}}$ $\left( i.e., P_{\mathbb {U}_{\textbf{n}}}\right)$ while maintaining the minimum threshold power at ${\mathbb {U}}_{\textbf{f}}$ such that $0< \alpha< \frac{1}{ \left( 1+ \max \left( {\bar{\gamma }}_{\textbf{f}},{\bar{\gamma }}_{\textbf{n}} \right) \right) }<1$.

#### Proof

Based on (3-5), (18) was derived such that $g _{\textbf{n}}={\eta }_{\textbf{f}}\left( \alpha \right) ={\psi }_{\textbf{n}}\left( \alpha \right)$. Thus, we have the following regions of $\alpha$:

When ${\eta }_{\textbf{f}}\left( \alpha \right) <{\psi }_{\textbf{n}}\left( \alpha \right)$, $0<\alpha \le \frac{{\bar{\gamma }} _{\textbf{n}}}{{\bar{\gamma }} _{\textbf{f}}{\bar{\gamma }} _{\textbf{n}}+{\bar{\gamma }} _{\textbf{f}}+ {\bar{\gamma }} _{\textbf{n}}}$, $P_{\mathbb {U}_{\textbf{n}}}=1- \mathbb {P} \left( g _{\textbf{n}}\ge \max \left( {\eta }_{\textbf{f}}\left( \alpha \right) ,{\psi }_{\textbf{n}}\left( \alpha \right) \right) \right) =1- \mathbb {P}\left( g _{\textbf{n}}\ge {\psi }_{\textbf{n}}\left( \alpha \right) \right) ={{F}_{g _{\textbf{n}}}}\left( {\psi }_{\textbf{n}}\left( \alpha \right) \right)$. From (7-8), it is observed that $P_{{\mathbb {U}}_{\textbf{n}}}$ is a monotonically decreasing function of $\alpha$ as ${{F}_{g _{\textbf{n}}}}\left( {\psi }_{\textbf{n}}\left( \alpha \right) \right)$ is a monotonically decreasing function of $\alpha$, i.e., $\frac{\partial P_{{\mathbb {U}}_{\textbf{n}}}}{\partial \alpha }<0$.

When ${\eta }_{\textbf{f}}\left( \alpha \right) >{\psi }_{\textbf{n}}\left( \alpha \right)$, $\frac{{\bar{\gamma }} _{\textbf{n}}}{{\bar{\gamma }} _{\textbf{f}}{\bar{\gamma }} _{\textbf{n}}+{\bar{\gamma }} _{\textbf{f}}+ {\bar{\gamma }} _{\textbf{n}}}<\alpha <\frac{1}{ \left( 1+ \max \left( {\bar{\gamma }}_{\textbf{f}},{\bar{\gamma }}_{\textbf{n}} \right) \right) }$, $P_{{\mathbb {U}}_{\textbf{n}}}=1- \mathbb {P}\left( g _{\textbf{n}}\ge {\eta }_{\textbf{f}}\left( \alpha \right) \right) ={{F}_{g _{\textbf{n}}}}\left( {\eta }_{\textbf{f}}\left( \alpha \right) \right)$. It is observed that $P_{{\mathbb {U}}_{\textbf{n}}}$ is a monotonically increasing function of $\alpha$ as ${{F}_{g _{\textbf{f}}}}\left( {\eta }_{\textbf{f}}\left( \alpha \right) \right)$ is a monotonically increasing function of $\alpha$, i.e., $\frac{\partial P_{{\mathbb {U}}_{\textbf{n}}}}{\partial \alpha }>0$. Furthermore, $g _{\textbf{f}}\le {\eta }_{\textbf{f}}\left( \alpha \right)$ is always satisfied whenever $g _{\textbf{n}}\le {\eta }_{\textbf{f}}\left( \alpha \right)$ is satisfied. Thus, the minimum threshold power can be achieved at ${\mathbb {U}}_{\textbf{f}}$ when $\alpha =\alpha _{min}$. $\square$

Substituting (18) into (12) and using (7), the system outage probability at $\alpha =\alpha _{min}$ can be expressed as

19 $$\begin{aligned} \begin{aligned} P_o =\left( 1- \mathbb {P}\left( {g _{\textbf{f}}\ge {\eta }_{\textbf{f}}\left( \alpha _{min}\right) ,g_{\textbf{n}}\ge {\psi }_{\textbf{n}}\left( \alpha _{min}\right) } \right) \right) ^M=\left( 1- \mathbb {P}\left( {g _{\textbf{f}}\ge \bar{{\eta }_{\textbf{f}}}} \right) \right) ^M=\left( \frac{\gamma \left( {{E_{\textbf{f}} },\frac{\sqrt{\bar{{\eta }_{\textbf{f}}}}}{{V_{\textbf{f}}\sqrt{U_{\textbf{f}}} }} } \right) }{\Gamma {\left( {{E_{\textbf{f}} }} \right) }}\right) ^M, \end{aligned} \end{aligned}$$

where $$\bar{{\eta }_{\textbf{f}}}={\frac{{\bar{\gamma }} _{\textbf{f}}{\bar{\gamma }} _{\textbf{n}}+{\bar{\gamma }} _{\textbf{f}}+ {\bar{\gamma }} _{\textbf{n}}}{\rho }}$$.

To solve the issue of joint antenna selection at BS and pairing of NOMA vehicular users, a greedy algorithm is provided in Algorithm 1 in which a best local decision is made at each step. Thus, it can offer a balance between performance and computational complexity.

### OMA

In the OMA scheme, spectrum sharing can be applied between the two users. As such, the detection probability requires that the events $\gamma _{\textbf{f}}^\star \ge {{2}^{\frac{{R_{f}}}{\left( 1-\delta \right) }}}-1$ and $\gamma _{\textbf{n}}^\star \ge {{2}^{\frac{{R_{n}}}{\delta }}}-1$ are jointly satisfied, where we adopt a power splitting mechanism such that $\gamma _{\textbf{f}}^\star =\rho \left( 1-\alpha \right) g_{\textbf{f}}$, $\gamma _{\textbf{n}}^\star =\rho \alpha g_{\textbf{n}}$, $\left( \textbf{n},\textbf{f} \right) \in \left\{ {\left( t,r \right) ,\left( r,t \right) } \right\}$ and $\delta$ is the sharing factor. The system outage performance can be derived by the following theorem.

#### Theorem 3

The system outage and asymptotic outage probability of ASRO scheme can be expressed as (11) and (16), respectively, where $\eta _{\textbf{f}}$ is replaced by ${\eta _{\textbf{f}}}^\star$, ${\eta _{\textbf{f}}}^\star =\frac{{{2}^{\frac{{R_{\textbf{f}}}}{\left( 1-\delta \right) }}}-1}{\rho \left( 1-\alpha \right) }$ and $\psi _{\textbf{n}}$ is replaced by ${\psi _{\textbf{n}}}^\star$, ${\psi _{\textbf{n}}}^\star =\frac{{{2}^{\frac{{R_{\textbf{n}}}}{\delta }}}-1}{\rho \alpha }$ for a fixed value of $\alpha$.

#### Remark 4

The outage behavior of ASRO scheme can also achieve a diversity order of *M*. However, the achievable coding gain is less than that of corresponding NOMA scheme, i.e., ASRN.

**Algorithm 1:**
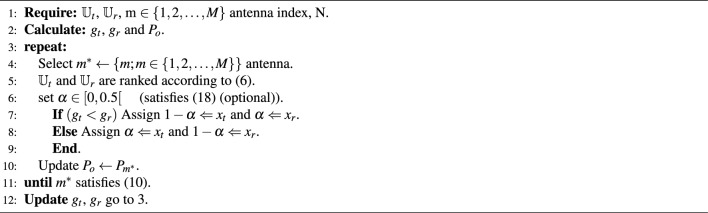
Joint antenna selection and NOMA pairing ASRN scheme.

## Ergodic sum rate

In this section, we investigate the ergodic sum rate (ESR) and the multiple averaging ergodic sum rate (MA-ESR) of the proposed ARSN scheme for arbitrary antenna selection at the BS and compare it to the cases of ASRO, CSRN and CSRO schemes. The ergodic sum rate (ESR) is a key performance metric that measures the average achievable data rate of all users in a communication system over fading channels representing a long-term average performance. However, averaging the ESR over multiple random variables describes the complex schemes that require the calculation of the joint statistics of the combined variables. In this sense, we define the MA-ESR to gain the expected performance of the proposed and comparative schemes. The MA-ESR is likely an ESR subjected to more than one layer of expectation over different sets of random variables to solve a complex system design.

For simplicity, let ${\eta }_{\textbf{f}}\left( x\right) = {\eta }_{\textbf{f}}\left( \alpha ,x\right)$, ${\psi }_{\textbf{n}}\left( x\right) ={\psi }_{\textbf{n}}\left( \alpha ,x\right)$, ${\eta }_{\textbf{f}}^{\star }\left( x\right) = {\eta }_{\textbf{f}}^{\star }\left( \alpha ,x\right)$ and ${\psi }_{\textbf{n}}^{\star }\left( x\right) ={\psi }_{\textbf{n}}^{\star }\left( \alpha ,x\right)$ for a specific value of $\alpha$.

### ESR

The ESR is defined as the expectation of the instantaneous sum rate. In the first, we define the ESR of the conventional schemes, i.e., CSRN and CSRO schemes. Then, it is used to determine the ESR of the proposed ASRN and comparative schemes under varying channel conditions. The ESR of the conventional schemes is given by the following definition.

#### Definition 1

The ESR of the conventional schemes^14^ is defined as

20 $$\begin{aligned}&{\mathbb {R}_{{\textbf{n}},{\textbf{f}}}^{ESR}} = {\mathbb {E}_{\gamma _{\textbf{n}}}} \left[ {\log _{2} \left( {1 + \gamma _{\textbf{n}}} \right) } \right] +{\mathbb {E}_{\gamma _{\textbf{f}}}} \left[ {\log _{2} \left( {1 + \gamma _{\textbf{f}}} \right) } \right] \nonumber = \int _0^\infty {{\log _{2} \left( {1 + x} \right) } {f_{{\gamma _{\textbf{n}}}}}\left( x \right) } dx + \int _0^\infty {{\log _{2} \left( {1 + y} \right) }{f_{{\gamma _{\textbf{f}}}}}\left( y \right) } dy \nonumber \\ &\overset{(\diamond )}{=}\ \frac{1}{\ln {2}} \left[ \int _0^\infty {\frac{1-{F_{{\gamma _{\textbf{n}}}}}\left( x \right) }{1+x} } dx + \int _0^\infty \frac{1-{F_{{\gamma _{\textbf{f}}}}}\left( y \right) }{1+y} dy \right] \end{aligned}$$

where $(\diamond )$ follows from integration by parts where ${\bar{F}}_{X} \left( \infty \right) =1-{F}_{X} \left( \infty \right) =0$ for a specific random variable *X*.

#### NOMA

Let ${\mathbb {P}_{\textbf{n},\textbf{f}}}=\mathbb {P}\left( g_{\textbf{n}}\ge g_{\textbf{f}} \right)$, $\textbf{n} \in \left\{ {t,r} \right\}$, $\textbf{f} \in \left\{ {\left\{ {t,r} \right\} -\left\{ {\textbf{n}} \right\} } \right\}$. Then, the ESR of the proposed ASRN scheme can be obtained based on **Definition** 1 by the following theorem.

##### Theorem 4

The ESR of the proposed ASRN scheme is expressed as

21 $$\begin{aligned} {\mathbb {R}_{ASRN}^{ESR}} = {\mathbb {P}_{\textbf{n},\textbf{f}}} {\mathbb {R}_{\textbf{n},\textbf{f}}^{ESR-N}} = {\mathbb {P}_{r,t}}{\mathbb {R}_{r,t}^{ESR-N}}+{\mathbb {P}_{t,r}}{\mathbb {R}_{t,r}^{ESR-N}}, \end{aligned}$$

where ${\mathbb {P}_{\textbf{n},\textbf{f}}}$ and ${\mathbb {R}_{\textbf{n},\textbf{f}}^{ESR-N}}$ are given by (22) and (23) at the top of the page, respectively; where $\textbf{n} \in \left\{ {t,r} \right\}$, $\textbf{f} \in \left\{ {\left\{ {t,r} \right\} -\left\{ {\textbf{n}} \right\} } \right\}$, ${\mathbb {P}_{r,t}}=1-{\mathbb {P}_{t,r}}$, ${x_k}= {y_k}= \cos \left( {\frac{{2k - 1}}{{2K}}\pi } \right)$, ${\omega _k} = \frac{\pi }{K}$, *K* is the convergent coefficient of the Chebyshev-Gauss-quadrature, $\chi _s\left( x_s\right) =\frac{x_s}{2\left( S+1\right) ^2L_{S+1}^2\left( x_s\right) }$ represents the weight of Gauss-Laguerre integration, *S* is the complexity accuracy tradeoff factor, $x_s$ is the *s*-th root of Laguerre polynomial $L_S\left( x_s\right)$ and ${\bar{F}}_{X} \left( x\right) =1-{F}_{X} \left( x\right)$.

22 $$\begin{aligned}&{\mathbb {P}_{\textbf{n},\textbf{f}}} = \frac{\Gamma \left( 2E\right) \left( {U_{\textbf{n}}}\right) ^{\frac{E}{2} -1}}{E \Gamma ^2 \left( E\right) } \left( \frac{\sqrt{{U_{\textbf{f}}} }}{\sqrt{{U_{\textbf{n}}} }+\sqrt{{U_{\textbf{f}}} }}\right) ^{2E} {_2F_1} \left( 1,2E;E+1;\frac{\sqrt{{U_{\textbf{f}}} }}{\sqrt{{U_{\textbf{n}}} }+\sqrt{{U_{\textbf{f}}} }} \right) . \end{aligned}$$

23 $$\begin{aligned} {\mathbb {R}_{\textbf{n},\textbf{f}}^{ESR-N}}&= \frac{1}{ \Gamma \left( E\right) \left( U_{\textbf{n}} \rho \alpha \right) ^E} G_{3,2}^{1,3}\Big ( { U_{\textbf{n}} \rho \alpha {V}^2} \Big \vert \begin{matrix} 1-E,1,1 \\ 1,0 \end{matrix} \Big ) \nonumber \\ &+ \frac{1 }{2 \Gamma \left( E\right) } \sum \limits _{k = 1}^K {\omega _k}\sqrt{1 - {y_k}^2} G_{2,2}^{1,2}\Big ( \frac{\left( 1-\alpha \right) y_k}{\alpha } \Big \vert \begin{matrix} 1,1 \\ 1,0 \end{matrix} \Big ) \frac{U_{\textbf{f}}}{\rho y_k \left( U_{\textbf{f}}-y_k \right) } \left( { \frac{\sqrt{y_k/{\rho \alpha \left( U_{\textbf{f}}-y_k\right) }} }{{V }}}\right) ^{E} {e^{ - \frac{\sqrt{y_k /{\rho \alpha \left( U_{\textbf{f}}-y_k\right) }} }{{V }}}}. \end{aligned}$$

##### Proof

Recall that ${{g}_{\mu }}={U_{\mu }}Z_{{\mu }}$, $\mu \in \left\{ {{\textbf{n}},{\textbf{f}}} \right\}$, ${U_{\mu }}=d_{b} ^{ - \zeta }d_{\mu } ^{ - \zeta }\lambda _{\mu }$; where $F_{g_{\mu }}\left( x\right) =F_{Z_{\mu }}\left( x/U_{\mu }\right)$ and $f_{g_{\mu }}\left( x\right) =1/U_{\mu } f_{Z_{\mu }}\left( x/U_{\mu }\right)$ are computed from (7) and (8), respectively. Then, ${\mathbb {P}_{\textbf{n},\textbf{f}}}$ can be obtained as

24 $$\begin{aligned}&{\mathbb {P}_{\textbf{n},\textbf{f}}} =\mathbb {P}\left( g_{\textbf{f}} < g_{\textbf{n}} \right) = \int _0^{\infty } {{F_{{g _{\textbf{f}}}}} \left( {x}\right) } {f_{{g_{\textbf{n}}}}} \left( {x}\right) dx = \frac{1}{{{U_{\textbf{n}}} }} \int _0^{\infty } {{F_{{Z _{\textbf{f}}}}} \left( \frac{x}{{U_{\textbf{f}}}}\right) } {f_{{Z _{\textbf{n}}}}} \left( \frac{x}{{U_{\textbf{n}}}}\right) dx \nonumber \\ &= \frac{1}{{{U_{\textbf{n}}} }} \int _0^{\infty } {\frac{\gamma \left( {{E_{{\textbf{f}}} },\frac{{\sqrt{x/{U_{\textbf{f}}}} }}{{{V_{{\textbf{f}}} }}}} \right) }{\Gamma {\left( {{E_{{\textbf{f}}} }} \right) }} \frac{{{{\left( \frac{x}{U_{\textbf{n}}}\right) }^{\frac{{E_{\textbf{n}} }}{2} - 1}}}}{{2 {\left( {V_{\textbf{n}}} \right) }^{E_{\textbf{n}} }\Gamma \left( {{{E_{\textbf{n}} }}} \right) }}{e^{ - \frac{\sqrt{x/{U_{\textbf{n}}}} }{{V_{\textbf{n}} }}}} } dx \overset{(a1)}{=}\ \frac{1}{\Gamma ^2 \left( E\right) \sqrt{{U_{\textbf{n}}} }} \int _0^\infty \gamma \left( E ,\sqrt{\frac{U_{\textbf{n}}}{U_{\textbf{f}}}} y \right) \left( \sqrt{{U_{\textbf{n}}}} y \right) ^{E-1} e^{-y} dy, \end{aligned}$$

where (*a*1) follows by putting $x= {U_{\textbf{n}}}\left( {V_{\textbf{n}}}y\right) ^2$, $E_{{\textbf{n}}}=E_{{\textbf{f}}}=E$, $V_{{\textbf{n}}}=V_{{\textbf{f}}}=V$, $U_{{\textbf{n}}} \ne U_{{\textbf{f}}}$ where $\left\{ \left( \textbf{n},\textbf{f} \right) \right\} \in \left\{ {\left( t,r \right) ,\left( r,t \right) } \right\}$. Then, (22) follows by invoking the product of an incomplete Gamma function and a Gamma-distributed PDF. This type of integral can be solved analytically using the identity in^26^, Eq. (6.455) to yield a closed-form expression involving the Gauss hypergeometric function.

Based on (20), the steps of obtaining ${\mathbb {R}_{\textbf{n},\textbf{f}}^{ESR-N}}$ can be found in (25) at the top of the page; where (*b*1) follows from the relationship between ${{g}_{\mu }}$ and ${{\gamma }_{\mu }}$, $\mu \in \left\{ {{\textbf{n}},{\textbf{f}}} \right\}$; and the boundary condition of power allocation coefficient, i.e., ${\eta }_{\textbf{f}}\left( y\right) : y \le \frac{1-\alpha }{\alpha }$, (*b*2) follows from the relationship between ${{Z}_{\mu }}$ and ${{g}_{\mu }}$, (*b*3) follows by forward substitution, putting $y= \frac{1-\alpha }{\alpha } l$ and interchanging variables, i.e., replacing *l* by *y*, (*b*4) follows by forward substitution, putting $x= {U_{\textbf{n}}} \rho \alpha \left( {V_{\textbf{n}}} l\right) ^2$ and interchanging variables, i.e., replacing *l* by *x*.

25 $$\begin{aligned}&{\mathbb {R}_{\textbf{n},\textbf{f}}^{ESR-N}} = \int _0^\infty {\log _{2} \left( {1 + x} \right) } f_{{\gamma _{\textbf{n}}}}\left( x \right) dx + \int _0^\infty {\log _{2} \left( {1 + x} \right) } f_{{\gamma _{\textbf{f}}}}\left( y \right) dy \nonumber \\&\overset{(b1)}{=}\ \frac{1}{{\ln 2}} \left[ \int _0^{\infty } \ln {\left( {1 + x} \right) } {\psi }^{'}_{\textbf{n}}\left( x\right) {f_{{g _{\textbf{n}}}}}\left( {\psi }_{\textbf{n}}\left( x\right) \right) dx+ \int _0^{\frac{1-\alpha }{\alpha }} \ln {\left( {1 + y} \right) } {\eta }^{'}_{\textbf{f}}\left( y\right) {f_{{g _{\textbf{f}}}}}\left( {\eta }_{\textbf{f}}\left( y\right) \right) dy \right] \nonumber \\&\overset{(b2)}{=}\ \frac{1}{{{U_{\textbf{n}}} \ln 2}} \int _0^{\infty } \ln {\left( {1 + x} \right) } {\psi }^{'}_{\textbf{n}}\left( \frac{x}{{U_{\textbf{n}}}}\right) {f_{{Z _{\textbf{n}}}}}\left( {\psi }_{\textbf{n}}\left( \frac{x}{{U_{\textbf{n}}}}\right) \right) dx+ \frac{1}{{{U_{\textbf{f}}} \ln 2}} \int _0^{\frac{1-\alpha }{\alpha }} \ln {\left( {1 + y} \right) } {\eta }^{'}_{\textbf{f}}\left( \frac{y}{{U_{\textbf{f}}}}\right) {f_{{Z _{\textbf{f}}}}}\left( {\eta }_{\textbf{f}}\left( \frac{y}{{U_{\textbf{f}}}}\right) \right) dy \nonumber \\&\overset{(b3)}{=}\frac{1}{{{U_{\textbf{n}} \rho \alpha \ln 2 } }} \int _0^{\infty } {\ln \left( 1+x\right) \frac{{{{\left( \frac{x}{U_{\textbf{n}} \rho \alpha }\right) }^{\frac{{E_{\textbf{n}} }}{2} - 1}}}}{{2 {\left( {V_{\textbf{n}}} \right) }^{E_{\textbf{n}} }\Gamma \left( {{{E_{\textbf{n}} }}} \right) }}{e^{ - \frac{\sqrt{x/{U_{\textbf{n}} \rho \alpha }} }{{V_{\textbf{n}} }}}} } dx \nonumber \\ &+ \frac{\left( 1-\alpha \right) }{\alpha {{U_{\textbf{f}}} \ln 2}} \int _0^{1} \ln {\left( {1 + \frac{\left( 1-\alpha \right) y}{\alpha }} \right) } {\eta }^{'}_{\textbf{f}}\left( \frac{\left( 1-\alpha \right) y}{\alpha {U_{\textbf{f}}}}\right) {f_{{Z _{\textbf{f}}}}}\left( {\eta }_{\textbf{f}}\left( \frac{\left( 1-\alpha \right) y}{\alpha {U_{\textbf{f}}}}\right) \right) dy \nonumber \\ &\overset{(b4)}{=}\ \frac{1}{\Gamma \left( E_{\textbf{n}}\right) \sqrt{{U_{\textbf{n}} \rho \alpha } }\ln 2} \int _0^\infty \ln \left( 1+{U_{\textbf{n}} \rho \alpha {V_{\textbf{n}}}}^2 x\right) \left( \sqrt{{U_{\textbf{n}} \rho \alpha }} x \right) ^{E_{\textbf{n}}-1} e^{-x} dx \nonumber \\ &+ \frac{1 }{2 \Gamma \left( E_{\textbf{f}}\right) \ln 2} \int _0^{1} \ln {\left( {1 + \frac{\left( 1-\alpha \right) y}{\alpha }} \right) } \frac{U_{\textbf{f}}}{\rho y \left( U_{\textbf{f}}-y\right) } \left( { \frac{\sqrt{y/{\rho \alpha \left( U_{\textbf{f}}-y\right) }} }{{V_{\textbf{n}} }}}\right) ^{E_{\textbf{f}}} {e^{ - \frac{\sqrt{y/{\rho \alpha \left( U_{\textbf{f}}-y\right) }} }{{V_{\textbf{n}} }}}} dy. \end{aligned}$$

Using the standard integral identity for the product of a Meijer-G function and a Gamma-distributed kernel^26^, Eq. (7.813), this integral can be expressed in closed form as a higher-order Meijer-G function. This approach is consistent with the methodology employed in^28^ with^30^, Eq. (2.24.1.3). Then, (23) follows by leveraging the relation $$\ln \left( 1+x \right) ={\ln 2} \times G_{2,2}^{1,2}\Big ( x \Big \vert \begin{matrix} 1,1 \\ 1,0 \end{matrix} \Big )$$ as in^31^ and applying the Gauss–Laguerre quadrature formula^32^ in the first integration; which is defined as $\int _0^\infty f\left( x\right) dx\approx \sum _{s=0}^{S} e^{x_s} \chi _s\left( x_s\right) f\left( x_s\right)$ where $\chi _s\left( x_s\right) =\frac{x_s}{2\left( S+1\right) ^2L_{S+1}^2\left( x_s\right) }$ represents the weight of Gauss-Laguerre integration, *S* is the complexity accuracy tradeoff factor, $x_s$ is the *s*-th root of Laguerre polynomial $L_S\left( x_s\right)$, and applying the Chebyshev-Gauss-quadrature formula in the second integration; which is defined as $\int _{ - 1}^1 {\frac{{f\left( y \right) }}{{\sqrt{1 - {y^2}} }}} dx \approx \sum \limits _{k = 1}^K {{\omega _k}f\left( {{y_k}} \right) }$ where ${\omega _k} = \frac{\pi }{K}$ and ${y_k} = \cos \left( {\frac{{2k - 1}}{{2K}}\pi } \right)$.

Finally, the ESR of the proposed ASRN scheme is obtained as (21) based on the condition of (6). $\square$

#### OMA

Regarding the spectrum sharing and the power splitting mechanism between the two users, the ESR of the ASRO scheme can be obtained based on **Definition** 1 by the following theorem.

##### Theorem 5

The ESR of the proposed ASRO scheme is expressed as

26 $$\begin{aligned} {\mathbb {R}_{ASRO}^{ESR}} = \frac{1}{2} {\mathbb {P}_{\textbf{n},\textbf{f}}} {\mathbb {R}_{\textbf{n},\textbf{f}}^{ESR-O}} = \frac{1}{2} \left( {\mathbb {P}_{r,t}}{\mathbb {R}_{r,t}^{ESR-O}}+{\mathbb {P}_{t,r}}{\mathbb {R}_{t,r}^{ESR-O}} \right) , \end{aligned}$$

where ${\mathbb {P}_{\textbf{n},\textbf{f}}}$ and ${\mathbb {R}_{\textbf{n},\textbf{f}}^{ESR-O}}$ are given by (22) and (27) at the top of the next page, respectively; where $\textbf{n} \in \left\{ {t,r} \right\}$, $\textbf{f} \in \left\{ {\left\{ {t,r} \right\} -\left\{ {\textbf{n}} \right\} } \right\}$, ${\mathbb {P}_{r,t}}=1-{\mathbb {P}_{t,r}}$, $\chi _s\left( x_s\right) =\frac{x_s}{2\left( S+1\right) ^2L_{S+1}^2\left( x_s\right) }$ represents the weight of Gauss-Laguerre integration, *S* is the complexity accuracy tradeoff factor, $x_s$ is the *s*-th root of Laguerre polynomial $L_S\left( x_s\right)$ and ${\bar{F}}_{X} \left( x\right) =1-{F}_{X} \left( x\right)$.

27 $$\begin{aligned} {\mathbb {R}_{\textbf{n},\textbf{f}}^{ESR-O}}&= \frac{1}{\Gamma \left( E\right) \sqrt{{U_{\textbf{n}} \rho \alpha } }} \sum \limits _{s = 1}^S \chi _s\left( x_s\right) G_{2,2}^{1,2}\Big ( {U_{\textbf{n}} \rho \alpha {V}}^2 x_s \Big \vert \begin{matrix} 1,1 \\ 1,0 \end{matrix} \Big ) \left( \sqrt{{U_{\textbf{n}} \rho \alpha }} x_s \right) ^{E-1} \nonumber \\ &+ \frac{1}{\Gamma \left( E\right) \sqrt{{U_{\textbf{f}} \rho \left( 1-\alpha \right) } }} \sum \limits _{s = 1}^S \chi _s\left( y_s\right) G_{2,2}^{1,2}\Big ( {U_{\textbf{f}} \rho \left( 1-\alpha \right) {V}}^2 y_s \Big \vert \begin{matrix} 1,1 \\ 1,0 \end{matrix} \Big ) \left( \sqrt{{U_{\textbf{f}} \rho \left( 1-\alpha \right) }} y_s \right) ^{E-1}. \end{aligned}$$

##### Proof

Recall again that $F_{g_{\mu }}\left( x\right) =F_{Z_{\mu }}\left( x/U_{\mu }\right)$, $f_{g_{\mu }}\left( x\right) =1/U_{\mu } f_{Z_{\mu }}\left( x/U_{\mu }\right)$, $\mu \in \left\{ {{\textbf{n}},{\textbf{f}}} \right\}$ and ${U_{\mu }}=d_{b} ^{ - \zeta }d_{\mu } ^{ - \zeta }\lambda _{\mu }$. Then, the steps of obtaining ${\mathbb {R}_{\textbf{n},\textbf{f}}^{ESR-O}}$ can be found in (28) based on (20) at the top of the page; where (*c*1) follows from the relationship between ${{g}_{\mu }}$ and ${{\gamma }_{\mu }}$, $\mu \in \left\{ {{\textbf{n}},{\textbf{f}}} \right\}$, (*c*2) follows from the relationship between ${{Z}_{\mu }}$ and ${{g}_{\mu }}$, (*c*3) follows by forward substitution. Then (27) follows by putting $x= {U_{\textbf{n}}} \rho \alpha \left( {V_{\textbf{n}}} l\right) ^2$ in the first integration, putting $y= {U_{\textbf{n}}} \rho \alpha \left( {V_{\textbf{n}}} l\right) ^2$ in the second integration, interchanging variables (i.e., replacing *l* by *x* and *l* by *y*, respectively) and applying the Gauss–Laguerre quadrature formula where $$\ln \left( 1+x \right) ={\ln 2} \times G_{2,2}^{1,2}\Big ( x \Big \vert \begin{matrix} 1,1 \\ 1,0 \end{matrix} \Big )$$.

Finally, the ESR of the proposed ASRO scheme is obtained as (26) based on the condition of (6); where $\left\{ \left( \textbf{n},\textbf{f} \right) \right\} \in \left\{ {\left( t,r \right) ,\left( r,t \right) } \right\}$, $E_{{t}}=E_{r}=E$ and $V_{{t}}=V_{r}=V$ while $U_{{t}} \ne U_{r}$ and the proof is completed. $\square$

28 $$\begin{aligned}&{\mathbb {R}_{\textbf{n},\textbf{f}}^{ESR-O}} = \int _0^\infty {\log _{2} \left( {1 + x} \right) } f_{{\gamma _{\textbf{n}}}}\left( x \right) dx + \int _0^\infty {\log _{2} \left( {1 + x} \right) } f_{{\gamma _{\textbf{f}}}}\left( y \right) dy \nonumber \\&\overset{(c1)}{=}\ \frac{1}{{\ln 2}} \left[ \int _0^{\infty } \ln {\left( {1 + x} \right) } {\psi }^{\star '}_{\textbf{n}}\left( x\right) {f_{{g _{\textbf{n}}}}}\left( {\psi }_{\textbf{n}}^{\star }\left( x\right) \right) dx+ \int _0^{\infty } \ln {\left( {1 + y} \right) } {\eta }^{\star '}_{\textbf{f}}\left( y\right) {f_{{g _{\textbf{f}}}}}\left( {\eta }_{\textbf{f}}^{\star }\left( y\right) \right) dy \right] \nonumber \\&\overset{(c2)}{=}\ \frac{1}{{{U_{\textbf{n}}} \ln 2}} \int _0^{\infty } \ln {\left( {1 + x} \right) } {\psi }^{\star '}_{\textbf{n}}\left( \frac{x}{{U_{\textbf{n}}}}\right) {f_{{Z _{\textbf{n}}}}}\left( {\psi }_{\textbf{n}}^{\star }\left( \frac{x}{{U_{\textbf{n}}}}\right) \right) dx+ \frac{1}{{{U_{\textbf{f}}} \ln 2}} \int _0^{\infty } \ln {\left( {1 + y} \right) } {\eta }^{\star '}_{\textbf{f}}\left( \frac{y}{{U_{\textbf{f}}}}\right) {f_{{Z _{\textbf{f}}}}}\left( {\eta }_{\textbf{f}}^{\star }\left( \frac{y}{{U_{\textbf{f}}}}\right) \right) dy \nonumber \\&\overset{(c3)}{=}\frac{1}{{{U_{\textbf{n}} \rho \alpha \ln 2 } }} \int _0^{\infty } {\ln \left( 1+x\right) \frac{{{{\left( \frac{x}{U_{\textbf{n}} \rho \alpha }\right) }^{\frac{{E_{\textbf{n}} }}{2} - 1}}}}{{2 {\left( {V_{\textbf{n}}} \right) }^{E_{\textbf{n}} }\Gamma \left( {{{E_{\textbf{n}} }}} \right) }}{e^{ - \frac{\sqrt{x/{U_{\textbf{n}} \rho \alpha }} }{{V_{\textbf{n}} }}}} } dx \nonumber \\ &+ \frac{1}{{{U_{\textbf{f}} \rho \left( 1- \alpha \right) \ln 2 } }} \int _0^{\infty } {\ln \left( 1+x\right) \frac{{{{\left( \frac{y}{U_{\textbf{f}} \rho \left( 1- \alpha \right) }\right) }^{\frac{{E_{\textbf{f}} }}{2} - 1}}}}{{2 {\left( {V_{\textbf{f}}} \right) }^{E_{\textbf{f}} }\Gamma \left( {{{E_{\textbf{f}} }}} \right) }}{e^{ - \frac{\sqrt{y/{U_{\textbf{f}} \rho \left( 1- \alpha \right) }} }{{V_{\textbf{f}} }}}} } dy. \end{aligned}$$

#### Asymptotic ESR

To further characterize the spectral efficiency in the high-SNR regime, we define the high-SNR slope (SL) of the ESR as $SL^{ESR} = \lim _{\rho \rightarrow \infty } \frac{\mathbb {R}^{ESR}_{\text {sum}}(\rho )}{\log _2(\rho )}$. Based on the asymptotic behavior of (3) and (4), as $\rho \rightarrow \infty$, $\gamma _{\textbf{f}}$ converges to a constant value $\frac{1-\alpha }{\alpha }$, resulting in a slope of $SL^{\gamma _{\textbf{f}}} = 0$. In contrast, $\gamma _{\textbf{n}}$ scales linearly with $\rho$, yielding a slope of $SL^{\gamma _{\textbf{n}}} = 1$. Consequently, the high-SNR slope of the proposed ASRN scheme is $SL_{ASRN}^{ESR} = SL^{\gamma _{\textbf{f}}} + SL^{\gamma _{\textbf{n}}} = 1$. This indicates that while STAR-RIS and TAS significantly shift the rate curves upward (improving the power offset/coding gain), the fundamental multiplexing gain of the two-user NOMA pair remains limited to a single stream due to the power-domain multiplexing over a single resource block.

At high transmit SNR, based on (3),(4) and **Definition** 1, the ESR of the proposed ASRN scheme can be asymptotically expressed as

29 $$\begin{aligned} {\mathbb {R}_{ASRN}^{ESR-Asy.}}&\approx {\mathbb {P}_{n,f}} \left[ \log _2 \left( 1+\frac{1-\alpha }{\alpha }\right) + \log _2(\rho ) +\log _2(\alpha g_{\textbf{n}})\right] \nonumber \\ &\approx {\mathbb {P}_{r,t}} \left[ \log _2 \left( 1+\frac{1-\alpha }{\alpha }\right) + \log _2(\rho ) +\log _2(\alpha g_{\textbf{r}})\right] + {\mathbb {P}_{t,r}} \left[ \log _2 \left( 1+\frac{1-\alpha }{\alpha }\right) + \log _2(\rho ) +\log _2(\alpha g_{\textbf{t}})\right] \nonumber \\ &\approx A \log _2(\rho ) + B, \end{aligned}$$

where $\log _2(1+\rho \alpha g) \approx \log _2(\rho \alpha g)$, ${\mathbb {P}_{r,t}}=1-{\mathbb {P}_{t,r}}$, *A* represents the high SNR slope (also known as the multiplexing gain or degrees of freedom) and *B* is a constant that captures the power offset. For the proposed ASRN scheme, $A = 1$ since the system achieves one degree of freedom through power-domain multiplexing. The constant *B* depends on the system parameters including the number of STAR-RIS elements *N*, the number of transmit antennas *M*, path loss exponents $\zeta$, distances, and power allocation coefficient $\alpha$.

##### Remark 5

The high-SNR slope analysis reveals several key insights:

- ${\mathbb {U}}_{\textbf{f}}$ is interference-limited at high SNR, with its SINR converging to $\frac{1-\alpha }{\alpha }$, resulting in a high-SNR slope of $SL^{\gamma _{\textbf{f}}} = 0$.
- ${\mathbb {U}}_{\textbf{n}}$ effectively removes the interference via SIC, allowing its rate to scale logarithmically with SNR, resulting in a high-SNR slope of $SL^{\gamma _{\textbf{n}}} = 1$.
- The ergodic sum rate slope of the system is therefore $SL_{ASRN}^{ESR} = 1$, which is consistent with the linear growth observed in the ESR vs. SNR plots.
- The primary benefit of the STAR-RIS and TAS integration lies not in increasing the multiplexing gain (slope), but in significantly enhancing the power offset (coding gain), which shifts the ESR curves to higher values compared to conventional OMA and CSRN schemes.
- Unlike the diversity order (which saturates at a finite value determined by *M* and *N*), the ESR continues to grow logarithmically with SNR without saturation, indicating that increasing transmit power always improves the average achievable rate.

For comparison, we consider an OMA scheme having a fraction $\Delta$ of the time/frequency assigned to ${\mathbb {U}}_{\textbf{n}}$ and the remaining fraction $1-\Delta$ of the time/frequency assigned to ${\mathbb {U}}_{\textbf{f}}$. Based on **Definition** 1, the ESR of the proposed ASRO scheme (i.e., ${\mathbb {R}_{ASRO}^{ESR}} ={\mathbb {P}_{n,f}}{\left[ (1-\Delta ) \log _2(1+\rho \alpha g_{\textbf{f}}/(1-\Delta ))+\Delta \log _2(1+\rho \alpha g_{\textbf{n}}/\Delta )\right] }$) can be asymptotically expressed as

30 $$\begin{aligned} {\mathbb {R}_{ASRO}^{ESR-Asy.}}&=\lim _{\rho \rightarrow \infty }{\mathbb {P}_{n,f}}{\left[ (1-\Delta ) \log _2(1+\rho \alpha g_{\textbf{f}}/(1-\Delta ))+\Delta \log _2(1+\rho \alpha g_{\textbf{n}}/\Delta )\right] } \nonumber \\ &\approx {\mathbb {P}_{r,t}}{\left[ (1-\Delta ) \log _2(\rho \alpha g_{\textbf{t}}/(1-\Delta ))+\Delta \log _2(\rho \alpha g_{\textbf{r}}/\Delta )\right] } \nonumber \\ &+{\mathbb {P}_{t,r}}{\left[ (1-\Delta ) \log _2(\rho \alpha g_{\textbf{r}}/(1-\Delta ))+\Delta \log _2(\rho \alpha g_{\textbf{t}}/\Delta )\right] }\nonumber \\ &\approx \log _2(\rho ) + \log _2(\alpha g_{\textbf{r}})+\log _2(\alpha g_{\textbf{t}})-(1-\Delta ) \log _2(1-\Delta )-\Delta \log _2(\Delta ). \end{aligned}$$

If $\Delta =1/2$, then ${\mathbb {R}_{ASRO}^{ESR}}$ can be rewritten as

31 $$\begin{aligned} {\mathbb {R}_{ASRO}^{ESR-Asy.}} \approx \log _2(\rho ) + \log _2(\alpha g_{\textbf{r}})+\log _2(\alpha g_{\textbf{t}})-1. \end{aligned}$$

For further comparison, we consider the case of the conventional cooperative NOMA/OMA (CC-NOMA/OMA) schemes^6^ where an intermediate relay supports communications to ${\mathbb {U}}_{\textbf{n}}$ and ${\mathbb {U}}_{\textbf{f}}$ in two time slots of operation. Based on the asymptotic behavior of the CC-NOMA scheme, $\gamma _{\textbf{f}}$ converges to a constant value $\frac{1-\alpha }{\alpha }$, whereas $\gamma _{\textbf{n}}$ converges to $\rho \alpha \min \left( g_{\textbf{R}},g_{\textbf{n}}\right)$ where $g_{\textbf{R}}$ is the channel gain from BS to the relay. The ESR of the CC-NOMA scheme can be expressed asymptotically as

32 $$\begin{aligned} {\mathbb {R}_{CC-NOMA}^{ESR-Asy.}} \approx \log _2 \left( 1+\frac{1-\alpha }{\alpha }\right) + \frac{1}{2}\log _2(\rho ) + \frac{1}{2}\log _2(\alpha g_{\textbf{n}})+ constant. \end{aligned}$$

##### Remark 6

The high-SNR slope analysis of ${\mathbb {R}_{ASRO}^{ESR-Asy.}}$ and ${\mathbb {R}_{CC-NOMA}^{ESR-Asy.}}$ reveal several key insights:

- The ergodic sum rate slope of ASRO is therefore $SL_{ASRO}^{ESR} = 1$, which is consistent with the linear growth observed in the ESR vs. SNR plots.
- $SL_{ASRN}^{ESR}$ and $SL_{ASRO}^{ESR}$ have the same ergodic sum rate slope but they have different coding gains.
- The ergodic sum rate slope of the CC-NOMA scheme is therefore $SL_{CC-NOMA}^{ESR} = 1/2$.
- Following the same concept as the CC-NOMA scheme, considering fractions of $\Delta$ and $1-\Delta$ are assigned to ${\mathbb {U}}_{\textbf{n}}$ and ${\mathbb {U}}_{\textbf{f}}$ as a spectrum sharing, the ergodic sum rate slope of the CC-OMA scheme is obtained as $SL_{CC-OMA}^{ESR} = 1/2$.

### MA-ESR

The MA-ESR is defined as the joint expectation of the instantaneous sum rate taking into account the variability of the wireless channels. We first define an analytical formula for the MA-ESR of the conventional schemes, i.e., CSRN and CSRO schemes. Then, closed-form expressions for the MA-ESR of the proposed ASRN and comparative schemes will be derived under varying channel conditions.

#### Definition 2

The MA-ESR of the conventional schemes is defined as

33 $$\begin{aligned} {\mathbb {R}_{{\textbf{n}},{\textbf{f}}}^{MA-ESR}} = {\mathbb {E}_{\gamma _{\textbf{n}},\gamma _{\textbf{f}}}} \left[ {\log _{2} \left( {1 + \gamma _{\textbf{n}}} \right) }+{\log _{2} \left( {1 + \gamma _{\textbf{f}}} \right) } \right] = \int _0^\infty {\int _0^\infty {{R_{\gamma _{\textbf{n}},\gamma _{\textbf{f}}}}\left( x,y \right) {f_{{\gamma _{\textbf{f}}}}}\left( x \right) {f_{{\gamma _{\textbf{n}}}}}\left( y \right) }} dx dy, \end{aligned}$$

where $${R_{\gamma _{\textbf{n}},\gamma _{\textbf{f}}}}\left( x,y \right) = \left[ {\log _{2} \left( {1 + x} \right) }+{\log _{2} \left( {1 + y} \right) } \right]$$.

The introduction of the MA-ESR metric is essential for accurately characterizing the stochastic coupling between users in the ASRN framework. While standard ESR is typically calculated as the sum of marginal expectations–treating users as independent links–MA-ESR performs a joint expectation over the ordered channel statistics–treating users as conditional links. In the proposed ASRN scheme, user identities are dynamically assigned based on the instantaneous condition $g_{\textbf{n}} > g_{\textbf{f}}$. Consequently, the achievable rate of each user is strictly conditioned on the state of the other. The ’multiple averaging’ effect refers to the simultaneous integration over the joint PDF of these coupled channels and the conditional boundaries of the SIC protocol. Unlike marginal ESR, which provides a theoretical capacity sum, MA-ESR represents the actual achievable protocol rate. From the allocation decision point of view at the BS, relying on standard ESR in low-to-medium SNR regimes would result in over-optimistic rate predictions; MA-ESR addresses this by accounting for the joint ranking logic and protocol constraints, providing a tighter and more realistic performance bound.

#### NOMA

The MA-ESR of the proposed ASRN scheme can be obtained based on **Definition** 2 by the following theorem.

##### Theorem 6

The MA-ESR of the proposed ASRN scheme is expressed as

34 $$\begin{aligned} {\mathbb {R}_{ASRN}^{MA-ESR}} = {\mathbb {P}_{\textbf{n},\textbf{f}}} {\mathbb {R}_{\textbf{n},\textbf{f}}^{MA-ESR-N}} = {\mathbb {P}_{r,t}}{\mathbb {R}_{r,t}^{MA-ESR-N}}+{\mathbb {P}_{t,r}}{\mathbb {R}_{t,r}^{MA-ESR-N}}, \end{aligned}$$

where ${\mathbb {P}_{\textbf{n},\textbf{f}}}$ and ${\mathbb {R}_{\textbf{n},\textbf{f}}^{MA-ESR-N}}$ are given by (22) and (35) at the top of the page, respectively; where $\textbf{n} \in \left\{ {t,r} \right\}$, $\textbf{f} \in \left\{ {\left\{ {t,r} \right\} -\left\{ {\textbf{n}} \right\} } \right\}$, ${x_k}= {y_k}= \cos \left( {\frac{{2k - 1}}{{2K}}\pi } \right)$, ${\omega _k} = \frac{\pi }{K}$, *K* is the convergent coefficient of the Chebyshev-Gauss-quadrature and ${\bar{F}}_{X} \left( x\right) =1-{F}_{X} \left( x\right)$.

35 $$\begin{aligned}&{\mathbb {R}_{\textbf{n},\textbf{f}}^{MA-ESR-N}} =\nonumber \\&\frac{\left( 1-\alpha \right) }{\alpha {{U_{\textbf{f}}} }} \sum \limits _{k = 1}^K {\omega _k}\sqrt{1 - {x_k}^2} {\bar{F}_{{Z _{\textbf{n}}}}}\left( {\psi }_{\textbf{n}}\left( \frac{\left( 1-\alpha \right) {x_k}}{\alpha {U_{\textbf{n}}}}\right) \right) G_{2,2}^{1,2}\Big ( \frac{\left( 1-\alpha \right) x_k}{\alpha } \Big \vert \begin{matrix} 1,1 \\ 1,0 \end{matrix} \Big ) {\eta }^{'}_{\textbf{f}}\left( \frac{\left( 1-\alpha \right) {x_k}}{\alpha {U_{\textbf{f}}}}\right) {f_{{Z _{\textbf{f}}}}}\left( {\eta }_{\textbf{f}}\left( \frac{\left( 1-\alpha \right) {x_k}}{\alpha {U_{\textbf{f}}}}\right) \right) \nonumber \\ &+ \frac{\left( 1-\alpha \right) }{\rho {\alpha ^2} {{U_{\textbf{n}}} }} \sum \limits _{k = 1}^K {\omega _k}\sqrt{1 - {y_k}^2} {{F_{{Z _{\textbf{f}}}}}\left( {\eta }_{\textbf{f}}\left( \frac{\left( 1-\alpha \right) {y_k}}{\alpha {U_{\textbf{f}}}}\right) \right) } G_{2,2}^{1,2}\Big ( \frac{\left( 1-\alpha \right) y_k}{\alpha } \Big \vert \begin{matrix} 1,1 \\ 1,0 \end{matrix} \Big ) {f_{{Z _{\textbf{n}}}}}\left( {\psi }_{\textbf{n}}\left( \frac{\left( 1-\alpha \right) {y_k}}{\alpha {U_{\textbf{n}}}}\right) \right) . \end{aligned}$$

##### Proof

Recall again that ${{g}_{\mu }}={U_{\mu }}Z_{{\mu }}$, $\mu \in \left\{ {{\textbf{n}},{\textbf{f}}} \right\}$, ${U_{\mu }}=d_{b} ^{ - \zeta }d_{\mu } ^{ - \zeta }\lambda _{\mu }$; where $F_{g_{\mu }}\left( x\right) =F_{Z_{\mu }}\left( x/U_{\mu }\right)$ and $f_{g_{\mu }}\left( x\right) =1/U_{\mu } f_{Z_{\mu }}\left( x/U_{\mu }\right)$ are computed from (7) and (8), respectively.

Based on (33), the steps of obtaining ${\mathbb {R}_{\textbf{n},\textbf{f}}^{MA-ESR-N}}$ can be found in (36) at the top of the next page; where (*d*1) follows from the double integration properties, (*d*2) follows from the relationship between ${{g}_{\mu }}$ and ${{\gamma }_{\mu }}$, $\mu \in \left\{ {{\textbf{n}},{\textbf{f}}} \right\}$; and the boundary condition of power allocation coefficient, i.e., $x<y\le \frac{1-\alpha }{\alpha } \Rightarrow \max \left( x,y\right) \le \frac{1-\alpha }{\alpha }$, (*d*3) follows from the relationship between ${{g}_{\mu }}$ and ${{Z}_{\mu }}$. Then (35) follows by applying the Chebyshev-Gauss-quadrature formula and using $$\ln \left( 1+x \right) ={\ln 2} \times G_{2,2}^{1,2}\Big ( x \Big \vert \begin{matrix} 1,1 \\ 1,0 \end{matrix} \Big )$$.

36 $$\begin{aligned}&{\mathbb {R}_{\textbf{n},\textbf{f}}^{MA-ESR-N}} \overset{(d1)}{=}\ \int _0^\infty {\int _0^\infty d{F_{{\gamma _{\textbf{n}}}}}\left( y \right) {\log _{2} \left( {1 + x} \right) } f_{{\gamma _{\textbf{f}}}}\left( x \right) dx } + \int _0^\infty {\int _0^\infty d{F_{{\gamma _{\textbf{f}}}}}\left( x \right) {\log _{2} \left( {1 + y} \right) } f_{{\gamma _{\textbf{n}}}}\left( y \right) dx } \nonumber \\&\overset{(d2)}{=}\ \frac{1}{{\ln 2}} \left[ \int _0^{\frac{1-\alpha }{\alpha }}{\int _x^\infty {d{F_{{g _{\textbf{n}}}}}\left( {\psi }_{\textbf{n}}\left( y\right) \right) \ln {\left( {1 + x} \right) }} d{F_{{g _{\textbf{f}}}}}\left( {\eta }_{\textbf{f}}\left( x\right) \right) } + \int _0^{\frac{1-\alpha }{\alpha }}{\int _0^y {d{F_{{g _{\textbf{f}}}}}\left( {\eta }_{\textbf{f}}\left( x\right) \right) } \ln {\left( {1 + y} \right) } d{F_{{g _{\textbf{n}}}}}\left( {\psi }_{\textbf{n}}\left( y\right) \right) } \right] \nonumber \\&= \frac{1}{{\ln 2}} \left[ \int _0^{\frac{1-\alpha }{\alpha }} {\bar{F}_{{g _{\textbf{n}}}}}\left( {\psi }_{\textbf{n}}\left( x\right) \right) \ln {\left( {1 + x} \right) } {\eta }^{'}_{\textbf{f}}\left( x\right) {f_{{g _{\textbf{f}}}}}\left( {\eta }_{\textbf{f}}\left( x\right) \right) dx + \int _0^{\frac{1-\alpha }{\alpha }} {{F_{{g _{\textbf{f}}}}}\left( {\eta }_{\textbf{f}}\left( y\right) \right) } \ln {\left( {1 + y} \right) } {\psi }^{'}_{\textbf{n}}\left( y\right) {f_{{g _{\textbf{n}}}}}\left( {\psi }_{\textbf{n}}\left( y\right) \right) dy \right] \nonumber \\&\overset{(d3)}{=}\ \frac{1}{{{U_{\textbf{f}}} \ln 2}} \int _0^{\frac{1-\alpha }{\alpha }} {\bar{F}_{{Z _{\textbf{n}}}}}\left( {\psi }_{\textbf{n}}\left( \frac{x}{{U_{\textbf{n}}}}\right) \right) \ln {\left( {1 + x} \right) } {\eta }^{'}_{\textbf{f}}\left( \frac{x}{{U_{\textbf{f}}}}\right) {f_{{Z _{\textbf{f}}}}}\left( {\eta }_{\textbf{f}}\left( \frac{x}{{U_{\textbf{f}}}}\right) \right) dx \nonumber \\ &+ \frac{1}{{{U_{\textbf{n}}} \ln 2}} \int _0^{\frac{1-\alpha }{\alpha }} {{F_{{Z _{\textbf{f}}}}}\left( {\eta }_{\textbf{f}}\left( \frac{y}{{U_{\textbf{f}}}}\right) \right) } \ln {\left( {1 + y} \right) } {\psi }^{'}_{\textbf{n}}\left( \frac{y}{{U_{\textbf{n}}}}\right) {f_{{Z _{\textbf{n}}}}}\left( {\psi }_{\textbf{n}}\left( \frac{y}{{U_{\textbf{n}}}}\right) \right) dy \nonumber \\&= \frac{\left( 1-\alpha \right) }{\alpha {{U_{\textbf{f}}} \ln 2}} \int _0^{1} {\bar{F}_{{Z _{\textbf{n}}}}}\left( {\psi }_{\textbf{n}}\left( \frac{\left( 1-\alpha \right) x}{\alpha {U_{\textbf{n}}}}\right) \right) \ln {\left( {1 + \frac{\left( 1-\alpha \right) x}{\alpha }} \right) } {\eta }^{'}_{\textbf{f}}\left( \frac{\left( 1-\alpha \right) x}{\alpha {U_{\textbf{f}}}}\right) {f_{{Z _{\textbf{f}}}}}\left( {\eta }_{\textbf{f}}\left( \frac{\left( 1-\alpha \right) x}{\alpha {U_{\textbf{f}}}}\right) \right) dx \nonumber \\ &+ \frac{\left( 1-\alpha \right) }{\alpha {{U_{\textbf{n}}} \ln 2}} \int _0^{1} {{F_{{Z _{\textbf{f}}}}}\left( {\eta }_{\textbf{f}}\left( \frac{\left( 1-\alpha \right) y}{\alpha {U_{\textbf{f}}}}\right) \right) } \ln {\left( {1 + \frac{\left( 1-\alpha \right) y}{\alpha }} \right) } {\psi }^{'}_{\textbf{n}}\left( \frac{\left( 1-\alpha \right) y}{\alpha {U_{\textbf{n}}}}\right) {f_{{Z _{\textbf{n}}}}}\left( {\psi }_{\textbf{n}}\left( \frac{\left( 1-\alpha \right) y}{\alpha {U_{\textbf{n}}}}\right) \right) dy. \end{aligned}$$

Finally, the ESR of the proposed ASRN scheme is obtained as (34) based on the condition of (6); where $\left\{ \left( \textbf{n},\textbf{f} \right) \right\} \in \left\{ {\left( t,r \right) ,\left( r,t \right) } \right\}$, $E_{{t}}=E_{r}=E$ and $V_{{t}}=V_{r}=V$ while $U_{{t}} \ne U_{r}$ and the proof is completed. $\square$

#### OMA

Regarding the spectrum sharing and the power splitting mechanism between the two users, the MA-ESR of the ASRO scheme can be obtained based on **Definition** 2 by the following theorem.

##### Theorem 1

The MA-ESR of the proposed ASRO scheme is expressed as

37 $$\begin{aligned} {\mathbb {R}_{ASRO}^{MA-ESR}} = \frac{1}{2} {\mathbb {P}_{\textbf{n},\textbf{f}}} {\mathbb {R}_{\textbf{n},\textbf{f}}^{MA-ESR-O}} = \frac{1}{2} \left( {\mathbb {P}_{r,t}}{\mathbb {R}_{r,t}^{MA-ESR-O}}+{\mathbb {P}_{t,r}}{\mathbb {R}_{t,r}^{MA-ESR-O}} \right) , \end{aligned}$$

where ${\mathbb {P}_{\textbf{n},\textbf{f}}}$ and ${\mathbb {R}_{\textbf{n},\textbf{f}}^{MA-ESR-O}}$ are given by (22) and (38) at the top of the next page, respectively; where $\textbf{n} \in \left\{ {t,r} \right\}$, $\textbf{f} \in \left\{ {\left\{ {t,r} \right\} -\left\{ {\textbf{n}} \right\} } \right\}$, $\chi _s\left( x_s\right) =\frac{x_s}{2\left( S+1\right) ^2L_{S+1}^2\left( x_s\right) }$ represents the weight of Gauss-Laguerre integration, *S* is the complexity accuracy tradeoff factor, $x_s$ is the *s*-th root of Laguerre polynomial $L_S\left( x_s\right)$ and ${\bar{F}}_{X} \left( x\right) =1-{F}_{X} \left( x\right)$.

38 $$\begin{aligned} {\mathbb {R}_{\textbf{n},\textbf{f}}^{MA-ESR-O}}&= \frac{1}{\rho \left( 1-\alpha \right) {U_{\textbf{f}}} } \sum \limits _{s = 1}^S e^{x_s} \chi _s\left( x_s\right) {\bar{F}_{{Z _{\textbf{n}}}}}\left( {\psi }_{\textbf{n}}^{\star }\left( \frac{x_s}{{U_{\textbf{n}}}}\right) \right) G_{2,2}^{1,2}\Big ( x_k \Big \vert \begin{matrix} 1,1 \\ 1,0 \end{matrix} \Big ) {f_{{Z _{\textbf{f}}}}}\left( {\eta }_{\textbf{f}}^{\star }\left( \frac{x_s}{{U_{\textbf{f}}}}\right) \right) \nonumber \\ &+ \frac{1}{\rho \alpha {U_{\textbf{n}}} } \sum \limits _{s = 1}^S e^{y_s} \chi _s\left( y_s\right) {{F_{{Z _{\textbf{f}}}}}\left( {\eta }_{\textbf{f}}^{\star }\left( \frac{y_s}{{U_{\textbf{f}}}}\right) \right) } G_{2,2}^{1,2}\Big ( y_k \Big \vert \begin{matrix} 1,1 \\ 1,0 \end{matrix} \Big ) {f_{{Z _{\textbf{n}}}}}\left( {\psi }_{\textbf{n}}^{\star }\left( \frac{y_s}{{U_{\textbf{n}}}}\right) \right) . \end{aligned}$$

##### Proof

Recall again that $F_{g_{\mu }}\left( x\right) =F_{Z_{\mu }}\left( x/U_{\mu }\right)$, $f_{g_{\mu }}\left( x\right) =1/U_{\mu } f_{Z_{\mu }}\left( x/U_{\mu }\right)$, $\mu \in \left\{ {{\textbf{n}},{\textbf{f}}} \right\}$ and ${U_{\mu }}=d_{b} ^{ - \zeta }d_{\mu } ^{ - \zeta }\lambda _{\mu }$.

Based on (33), the steps of obtaining ${\mathbb {R}_{\textbf{n},\textbf{f}}^{MA-ESR-O}}$ can be found in (39) at the top of the page; where (*e*1) follows from the double integration properties and the relationship between ${{g}_{\mu }}$ and ${{\gamma }_{\mu }}$, $\mu \in \left\{ {{\textbf{n}},{\textbf{f}}} \right\}$; (*e*2) follows from the relationship between ${{g}_{\mu }}$ and ${{Z}_{\mu }}$. Then (38) follows by applying the Gauss–Laguerre quadrature formula and using $$\ln \left( 1+x \right) ={\ln 2} \times G_{2,2}^{1,2}\Big ( x \Big \vert \begin{matrix} 1,1 \\ 1,0 \end{matrix} \Big )$$.

39 $$\begin{aligned}&{\mathbb {R}_{\textbf{n},\textbf{f}}^{MA-ESR-O}} = \int _0^\infty {\int _0^\infty d{F_{{\gamma _{\textbf{n}}}}}\left( y \right) {\log _{2} \left( {1 + x} \right) } f_{{\gamma _{\textbf{f}}}}\left( x \right) dx } + \int _0^\infty {\int _0^\infty d{F_{{\gamma _{\textbf{f}}}}}\left( x \right) {\log _{2} \left( {1 + y} \right) } f_{{\gamma _{\textbf{n}}}}\left( y \right) dx } \nonumber \\&\overset{(e1)}{=}\ \frac{1}{{\ln 2}} \left[ \int _0^\infty {\int _x^\infty {d{F_{{g _{\textbf{n}}}}}\left( {\psi }_{\textbf{n}}^{\star }\left( y\right) \right) \ln {\left( {1 + x} \right) }} d{F_{{g _{\textbf{f}}}}}\left( {\eta }_{\textbf{f}}^{\star }\left( x\right) \right) } dx + \int _0^\infty {\int _0^y {d{F_{{g _{\textbf{f}}}}}\left( {\eta }_{\textbf{f}}^{\star }\left( x\right) \right) } \ln {\left( {1 + y} \right) } d{F_{{g _{\textbf{n}}}}}\left( {\psi }_{\textbf{n}}^{\star }\left( y\right) \right) } dy \right] \nonumber \\&= \frac{1}{{\ln 2}} \left[ \int _0^\infty {\bar{F}_{{g _{\textbf{n}}}}}\left( {\psi }_{\textbf{n}}^{\star }\left( x\right) \right) \ln {\left( {1 + x} \right) } {\eta }^{\star '}_{\textbf{f}}\left( x\right) {f_{{g _{\textbf{f}}}}}\left( {\eta }_{\textbf{f}}^{\star }\left( x\right) \right) dx + \int _0^\infty {{F_{{g _{\textbf{f}}}}}\left( {\eta }_{\textbf{f}}^{\star }\left( y\right) \right) } \ln {\left( {1 + y} \right) } {\psi }^{\star '}_{\textbf{n}}\left( y\right) {f_{{g _{\textbf{n}}}}}\left( {\psi }_{\textbf{n}}^{\star }\left( y\right) \right) dy \right] \nonumber \\&\overset{(e2)}{=}\ \frac{1}{{{U_{\textbf{f}}} \ln 2}} \int _0^\infty {\bar{F}_{{Z _{\textbf{n}}}}}\left( {\psi }_{\textbf{n}}^{\star }\left( \frac{x}{{U_{\textbf{n}}}}\right) \right) \ln {\left( {1 + x} \right) } {\eta }^{\star '}_{\textbf{f}}\left( \frac{x}{{U_{\textbf{f}}}}\right) {f_{{Z _{\textbf{f}}}}}\left( {\eta }_{\textbf{f}}^{\star }\left( \frac{x}{{U_{\textbf{f}}}}\right) \right) dx \nonumber \\ &+ \frac{1}{{{U_{\textbf{n}}} \ln 2}} \int _0^\infty {{F_{{Z _{\textbf{f}}}}}\left( {\eta }_{\textbf{f}}^{\star }\left( \frac{y}{{U_{\textbf{f}}}}\right) \right) } \ln {\left( {1 + y} \right) } {\psi }^{\star '}_{\textbf{n}}\left( \frac{y}{{U_{\textbf{n}}}}\right) {f_{{Z _{\textbf{n}}}}}\left( {\psi }_{\textbf{n}}^{\star }\left( \frac{y}{{U_{\textbf{n}}}}\right) \right) dy. \end{aligned}$$

Finally, the ESR of the proposed ASRO scheme is obtained as (37) based on the condition of (6); where $\left\{ \left( \textbf{n},\textbf{f} \right) \right\} \in \left\{ {\left( t,r \right) ,\left( r,t \right) } \right\}$, $E_{{t}}=E_{r}=E$ and $V_{{t}}=V_{r}=V$ while $U_{{t}} \ne U_{r}$ and the proof is completed. $\square$

##### Remark 7

In vehicular NOMA systems, the MA-ESR is an efficient tool in comparison to the standard ESR to characterize the performance of the ergodic rate of those complex systems involving multiple random variables that are conditioned on each other in order to understand performance across the entire system. While the standard ESR provides a simplified performance benchmark by summing the individual average rates of users, it often ignores the underlying statistical coupling between them. In contrast, the proposed MA-ESR captures the joint variability of both NOMA users’ channels simultaneously. Because user identities in ASRN are dynamically assigned based on instantaneous CSI, their rates are statistically coupled. MA-ESR integrates the joint PDF over the specific regions where the gain-adaptive logic and SIC decoding constraints are simultaneously satisfied, providing a more realistic performance measure than marginal ESR. By utilizing the joint probability density function (PDF), MA-ESR reflects the real-world behavior of delay-tolerant vehicular networks, where the BS must account for the simultaneous fading states of multiple users to maintain the NOMA power-domain hierarchy and ensure successful SIC. More specifically, Eqs. (35) and (38) represent the probability of occurrence of the event that $\mathbb {U}_t$ and $\mathbb {U}_r$ become near and far users and vice versa, while Eqs. (36) and (39) represent the joint ergodic statistics of the sum rate under this condition for both NOMA and OMA cases, respectively.

##### Remark 8

The ESR performance is regarded as a tight upper bound for the true performance, i.e., the complex MA-ESR performance, which lies in decoupling the expectation over the multiple random variables. This approximation transforms a complex joint averaging into a sum of more tractable individual ergodic rates to describe the performance of the proposed ASRN and comparative schemes in delay-tolerant network mode, regarding the variability of the wireless communications’ channels.

## Numerical results

In this section, we provide the numerical results to verify the analytical work via Monte Carlo simulation with ${{10}^{5}}$ iterations. Furthermore, we investigate the benefits of dynamic gain adaptation in the performance enhancement of ASRN/ASRO over CSRN/CSRO schemes with both arbitrary^6,10,11^ and optimal^29^ fixed power reallocation mechanisms where the impact of increasing the transmit SNR ($\rho$), the number of RIS elements (*N*) and the relative distance between the two vehicular users with respect to STAR-RIS is also illustrated. Without loss of generality, the main parameters are specified as follows. For example, we set^11^ $R_{t}=R_{r}=1$ bit/channel use (BPCU), $\alpha =0.2$, $N=100$, $M=10$, $\zeta =2.5$, ${d_{b}}=5$ *m* and $\kappa =-5$ *dB*. In fact, the choice of values of $\zeta$ and $\kappa$ is intended to model a challenging urban micro-cell environment. A lower path loss exponent is consistent with RIS-assisted links that benefit from optimized surface placement. A negative Rician factor (in *dB*) represents a high-scattering scenario with partially obstructed line-of-sight. Furthermore, while the distances are set to model a roadside STAR-RIS deployment, the analytical derivations provided in this work are scale-invariant and applicable to larger vehicular deployment scenarios.

To isolate the benefits of the proposed adaptations, we compare ASRN against several critical baselines. Specifically, CSRN represents the fixed-SIC-ranking baseline with fixed power allocation. A ’NOMA without RIS’ baseline is not included here as it results in total system outage under the heavy-blockage vehicular environment, i.e., the direct gain to users is blocked ($g_{direct}=0$) as assumed in this work.

**Fig. 2:**
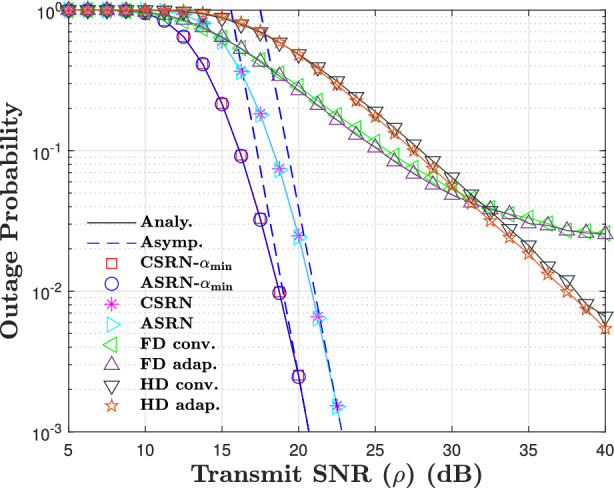
Outage performance of ASRN-$\alpha _{min}$, ASRN, CSRN-$\alpha _{min}$, CSRN and benchmark schemes vs. $\rho$ where ${d_{r}}=6$ *m* and ${d_{t}}=12$ *m*.

In Fig. 2, we investigate the impact of $\alpha _{min}$ on the system outage probability of the ASRN compared to the CSRN schemes and the half-duplex and full-duplex (HD/FD) DF relay-assisted NOMA schemes in terms of transmit SNR ($\rho$) when $\left( \textbf{n},\textbf{f} \right) =\left( r,t \right)$, i.e., for example, ${d_{r}}=6$ *m* and ${d_{t}}=12$ *m*. The analytical curves of ASRN schemes are plotted according to (11) and (16) at $\alpha =0.2$ and $\alpha _{min}=0.3333$ (denoted by ASRN-$\alpha _{min}$) while the analytical curves of CSRN schemes are plotted according to^11^, Eq. 19 and (19) (denoted by CSRN-$\alpha _{min}$), respectively. Obviously, the analytical curves precisely match with simulation points which verifies the correctness of our derivations. It is also observed that the outage performance of ASRN schemes slightly coincide with CSRN schemes. The reason is that $\mathbb {U}_t$ is still considered as a far user and $\mathbb {U}_r$ performs SIC as a near user as well as CSRN schemes. Furthermore, the outage performance of ASRN-$\alpha _{min}$/CSRN-$\alpha _{min}$ schemes are better than ASRN/CSRN and HD/FD DF relay-assisted NOMA schemes^6,10^ due to the optimal choice of the power allocation coefficient $\alpha _{min}$ for decoding order at BS.

**Fig. 3:**
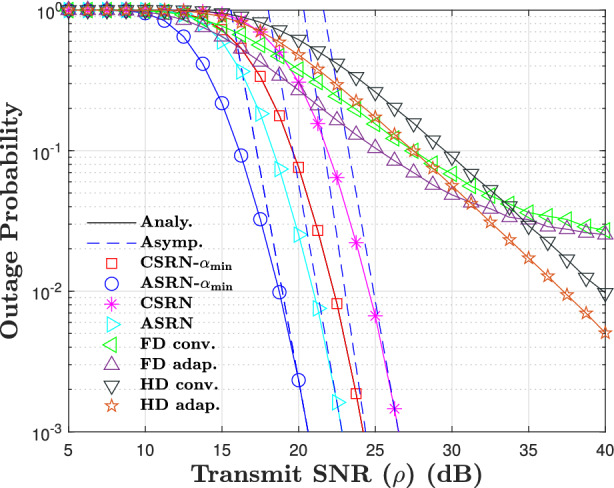
Outage performance of ASRN-$\alpha _{min}$, ASRN, CSRN-$\alpha _{min}$, CSRN and benchmark schemes vs. $\rho$ where ${d_{r}}=12$ *m* and ${d_{t}}=6$ *m*.

The benefit of dynamic gain-adaptive scheme is highlighted in Fig. 3 where $\left( \textbf{n},\textbf{f} \right) =\left( t,r \right)$, i.e., ${d_{t}}=6$ *m* and ${d_{r}}=12$ *m*. In such a case, $\mathbb {U}_t$ is considered as a near user and adapted to perform SIC in ASRN schemes while $\mathbb {U}_r$ still indeed performs SIC in CSRN schemes. The outage probability of ASRN schemes are remarkably better than CSRN schemes. Furthermore, ASRN-$\alpha _{min}$ scheme outperforms ASRN due to the adaptation with optimal power coefficient. In addition, they are all better than HD/FD DF cooperative relay assisted NOMA schemes. On one hand, ASRN schemes guarantee better fairness for users by SIC ranking and power coefficient reallocation in comparison of CSRN schemes. On the other hand, FD DF relay assisted NOMA schemes suffer from delay processing and residual self-interference in the FD mechanism while it provides better utilization of resources than HD DF relay NOMA schemes.

**Fig. 4:**
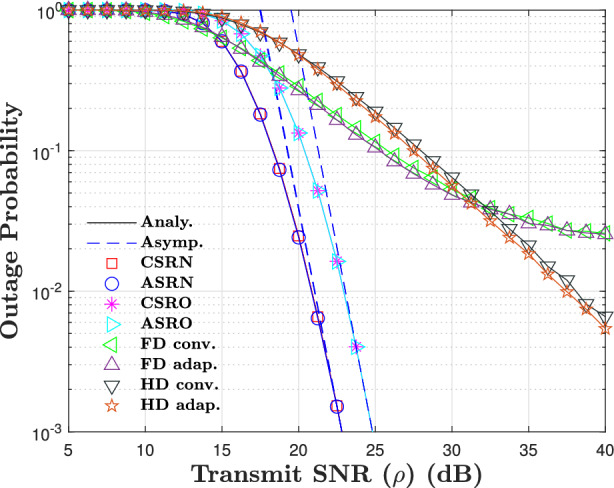
Outage performance of ASRN, ASRO, CSRN, CSRO and benchmark schemes vs. $\rho$ where ${d_{r}}=6$ *m* and ${d_{t}}=12$ *m*.

To make a performance comparison with the OMA cases, the system outage probability of ASRN in comparison with CSRN^11^, ASRO, CSRO and HD/FD DF cooperative relay assisted NOMA adaptive and conventional schemes is illustrated in Fig. 4 in terms of transmit SNR ($\rho$) when $\left( \textbf{n},\textbf{f} \right) =\left( r,t \right)$, i.e., for instance, ${d_{r}}=6$ *m* and ${d_{t}}=12$ *m*. The analytical curves of ASRO schemes are plotted based on **Theorem** 2 by substituting in (11) and (16) whereas the analytical curves of CSRO schemes are plotted according to^11^, Eq. 19 based on **Theorem** 2 without direct links between BS and users. It is evident that the analytical curves precisely match with simulation points which verifies the correctness of our derivations. In addition, the outage performance of adaptive schemes slightly outperform or coincide with conventional schemes which are better than HD/FD DF relay assisted NOMA schemes.

**Fig. 5:**
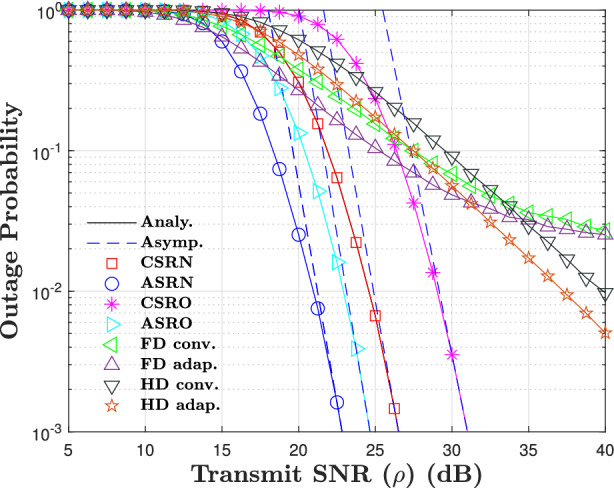
Outage performance of ASRN, ASRO, CSRN, CSRO and benchmark schemes vs. $\rho$ where ${d_{r}}=12$ *m* and ${d_{t}}=6$ *m*.

However, in Fig. 5 where $\left( \textbf{n},\textbf{f} \right) =\left( t,r \right)$, i.e., ${d_{t}}=6$ *m* and ${d_{r}}=12$ *m*. In such a case, $\mathbb {U}_t$ is considered to be a near user and adapted to perform SIC whereas $\mathbb {U}_r$ still indeed performs SIC in conventional schemes. The outage probability of ASRN and ASRO schemes are remarkably better than CSRN and CSRO schemes, respectively. Besides, they are better than HD/FD DF cooperative relay assisted NOMA schemes. Furthermore, ASRN scheme provides better adaptation by SIC and power allocation re-ordering in comparison of CSRN scheme whereas it achieves better user fairness than ASRO scheme.

**Fig. 6:**
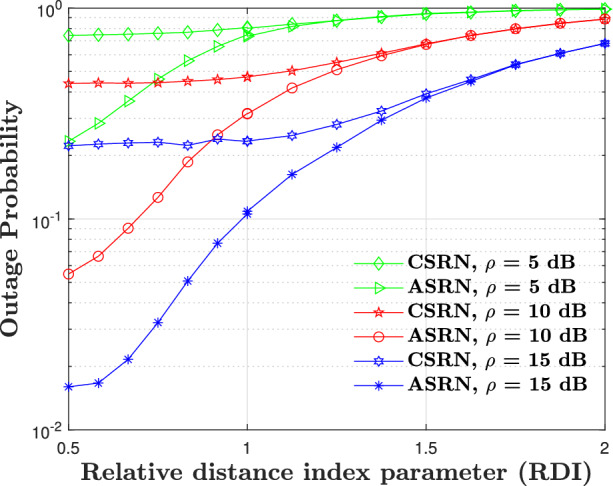
Outage performance comparison between ASRN scheme and CSRN scheme vs. *RDI* for different values of $\rho$.

Let us define the relative distance index parameter as $\left( RDI={{d}_{t}}/{{d}_{r}} \right)$ in which $\left( \textbf{n},\textbf{f} \right) =\left( t,r \right)$ for $RDI\in \left[ 0,1 \right]$ and $\left( \textbf{n},\textbf{f} \right) =\left( r,t \right)$ where $RDI\in \left[ 1,\infty \right]$. In Fig. 6, the system outage probability is plotted against *RDI* as $\rho = 5$, 10 and 15 *dB*. It is observed that the system outage performance of ASRN scheme becomes better than CSRN scheme in the region of $RDI\in \left[ 0,1 \right]$. The performance gap increases as $\rho$ rises and decreases with the increase of *RDI* until the two outage performances coincide when *RDI* converges to $\infty$.

**Fig. 7:**
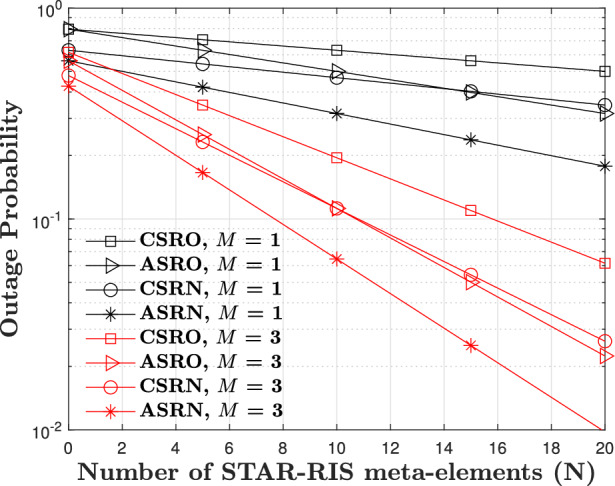
Outage performance comparison of ASRN, ASRO, CSRN and CSRO schemes vs. *N*.

The impact of increasing *N* is illustrated in Fig. 7 with $\rho =10$ dB and $M=1,3$. When $\left( \textbf{n},\textbf{f} \right) =\left( t,r \right)$, the system outage performance of all schemes is significantly improved with increasing *N* where *M* adds another degree of freedom. Moreover, the system outage performance of ASRN and ASRO schemes are always much better than CSRN^29^ and CSRO schemes^11^, respectively.

Considering an arbitrary selection of the BS transmit antenna, the calculation of the MA-ESR is an appropriate tool to investigate the impact of the proposed ASRN scheme in delay-tolerant regime. The ESR and MA-ESR performance of ASRN, CSRN, ASRO and CSRO are illustrated in the following figures. For further performance comparison, CC-NOMA/OMA relaying schemes are added as a benchmark comparison to illustrate the benefits of deploying STAR-RIS components over conventional relaying^33,34^.

**Fig. 8:**
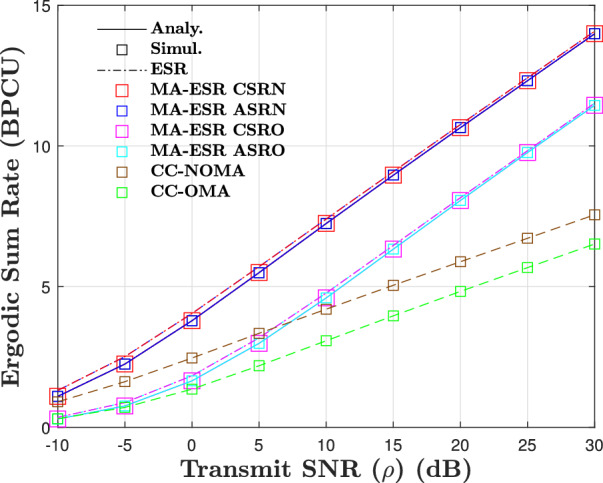
MA-ESR of ASRN, ASRO, CSRN, CSRO and CC-NOMA/OMA schemes vs. $\rho$ where ${d_{r}}=12$ *m* and ${d_{t}}=6$ *m*..

Firstly, both ESR and MA-ESR performances of ASRN scheme in comparison with ASRO, CSRN and CSRO schemes are illustrated in Fig. 8 in terms of transmit SNR ($\rho$) when $\left( \textbf{n},\textbf{f} \right) =\left( r,t \right)$, i.e., for instance, ${d_{r}}=6$ *m* and ${d_{t}}=12$ *m*. The ESR of ASRN and ASRO schemes are plotted based on **Theorem** 3 and **Theorem** 4, respectively; the MA-ESR of ASRN and ASRO schemes are plotted based on **Theorem** 5 and **Theorem** 6, respectively; whereas CSRN and CSRO schemes are plotted based on **Definition** 1 and **Definition** 2 by straight forward substitution in (20) and (33) without and with spectrum sharing, respectively. It is observed that the MA-ESR of adaptive schemes (i.e., ASRN and ASRO) slightly outperform or coincide with conventional schemes (i.e., CSRN and CSRO) while the MA-ESR performance of ASRN scheme is better than that of ASRO scheme. Moreover, the ESR performance represents a tight upper bound to the MA-ESR performance where the MA-ESR curves of all schemes converge to the ESR curves with the increase of transmit SNR, i.e., $\rho \rightarrow \infty$.

On the other hand, results demonstrate that ASRN exhibit significantly superior ESR compared to CC-NOMA/OMA schemes that relies on standard relays. The ability to independently tune energy splitting (ES) for cell-center (reflected) and cell-edge (transmitted) users maximizes the achievable rate without requiring the active; power-hungry RF chains needed by relays. Additionally, the CC-NOMA/OMA schemes typically offer a lower slope due to half-duplex losses or limited relay power compared to the “passive beamforming” gain of all STAR-RIS assisted schemes.

**Fig. 9:**
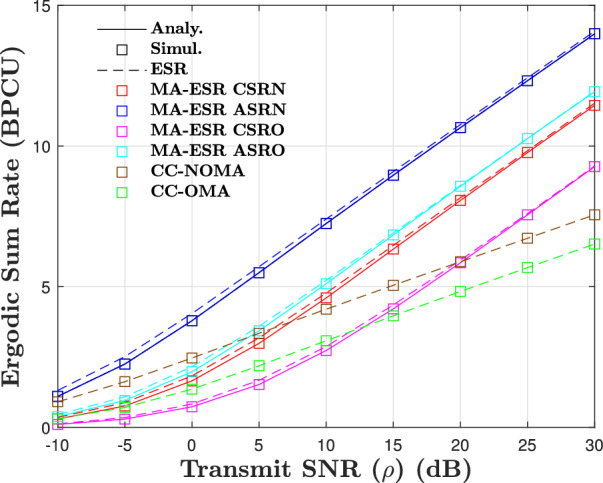
MA-ESR of ASRN, ASRO, CSRN, CSRO and CC-NOMA/OMA schemes vs. $\rho$ where ${d_{r}}=6$ *m* and ${d_{t}}=12$ *m*.

**Fig. 10:**
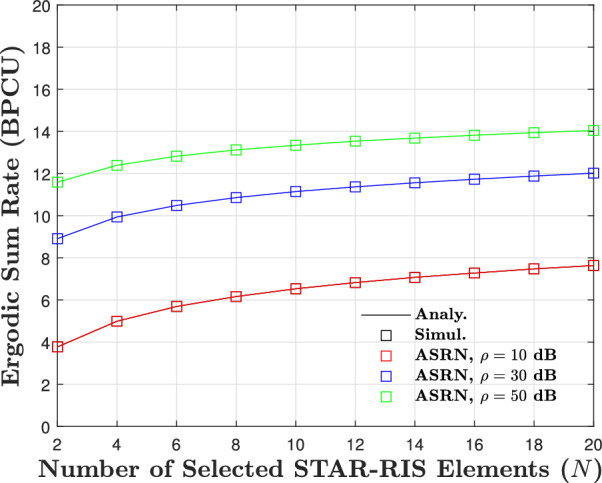
MA-ESR of ASRN, ASRO, CSRN and CSRO schemes vs. *N* where ${d_{r}}=12$ *m* and ${d_{t}}=6$ *m*.

However, in Fig. 9 where $\left( \textbf{n},\textbf{f} \right) =\left( t,r \right)$, i.e., ${d_{t}}=6$ *m* and ${d_{r}}=12$ *m*. It is observed that both ESR and MA-ESR performances of both ASRN and ASRO schemes are remarkably better than that of both CSRN and CSRO schemes, respectively; while the ASRN scheme has the best performance than others. The reason is that ASRN scheme provides better adaptation of $\alpha$ compared to CSRN scheme where the MA-ESR is directly proportional to $\alpha$. In addition, ASRN scheme achieves better user fairness than ASRO scheme. Furthermore, the MA-ESR performances all schemes represent a tight upper bound and converge to the ESR performances as $\rho \rightarrow \infty$. It is observed in Figs. 8, 9 and 10 that MA-ESR converges to the standard ESR at high SNR. This convergence indicates that at high transmit power, the stochastic coupling of the users due to channel ranking becomes negligible. However, in the low-to-medium SNR regime, MA-ESR yields a tighter, more realistic achievable rate bound than ESR. This is because MA-ESR accounts for the joint protocol constraints of the STAR-RIS refracted and reflected links, making it a vital metric for accurate resource allocation in power-constrained vehicular environments. It can be further confirmed that STAR-RIS-assisted NOMA networks achieve the highest ESR when compared to conventional relaying, and standard Orthogonal Multiple Access (OMA) baselines. The spatial flexibility to serve users on both sides of the surface ensures reliable transmission environments that conventional relays physically cannot match. In addition, ASRN scheme consistently outperforms the CC-NOMA relaying scheme, the gain becomes more significant at medium–high $\rho$ and the ASRN scheme exhibits a double steeper slope than the CC-NOMA/OMA relaying schemes at very large values of $\rho$.

In Fig. 10, the MA-ESR of ASRN scheme is plotted for different values of *N*. It is observed that the slopes of the curves are fixed in high $\rho$ regions which reflects that the scale of MA-ESR of ASRN scheme is independent of the number of STAR-RIS elements, i.e., *N*, for high values of $\rho$ (i.e., the gain from increasing STAR-RIS elements becomes smaller).

## Conclusion

The proposed dynamic gain-adaptive antenna selection scheme demonstrates superior performance for vehicular NOMA networks, particularly in high-mobility scenarios where channel conditions vary. The current analysis assumes quasi-static Rician fading to establish tractable closed-form performance bounds. Future extensions may incorporate Doppler shift, channel temporal correlation, and imperfect CSI estimation to further evaluate the robustness of the proposed ASRN scheme under more extreme high-mobility conditions. The current analytical and simulation results show that the proposed scheme achieves significant outage performance and coding gain over conventional STAR-RIS NOMA/OMA and DF relaying schemes, with improved diversity and coding gains. In case of delay-tolerant mode, we define MA-ESR which is generally more robust performance evaluation that accounts for the combined effects of different channel conditions, power allocation, and other system parameters. The ESR performance represents a tight upper bound to the MA-ESR performance with less computational complexity. The proposed ASRN scheme has the ability to dynamically adapt to channel variations while maintaining the performance target, making it a practical solution for future vehicular communication systems.

## Data Availability

The dataset used and/or analyzed during this study is available from the corresponding author upon a reasonable request.

## Footnotes

1 The two NOMA users model was previously considered to reduce the complexity^5^.

## References

[1] Basharat, S. et al. Reconfigurable intelligent surfaces: Potentials, applications, and challenges for 6G wireless networks. *IEEE Wirel. Commun.* **28**, 184–191 (2021).

[2] Liu, Y. et al. Reconfigurable intelligent surfaces: Principles and opportunities. *IEEE Commun. Surv. Tutorials* **23**, 1546–1577 (2021).

[3] Liu, Y. et al. STAR: Simultaneous transmission and reflection for coverage by intelligent surfaces. *IEEE Wirel. Commun.* **28**, 102–109 (2022).

[4] Mu, X., Liu, Y., Guo, L., Lin, J. & Schober, R. Simultaneously transmitting and reflecting (STAR) RIS aided wireless communications. *IEEE Trans. Wireless Commun.* **21**, 3083–3098 (2021).

[5] Ding, Z., Fan, P. & Poor, H. V. Impact of user pairing on 5G nonorthogonal multiple-access downlink transmissions. *IEEE Trans. Veh. Technol.* **65**, 6010–6023 (2015).

[6] Ding, Z., Dai, H. & Poor, H. V. Relay selection for cooperative NOMA. *IEEE Wirel. Commun. Lett.* **5**, 416–419 (2016).

[7] Li, Y., Li, Y., Chu, X., Ye, Y. & Zhang, H. Performance analysis of relay selection in cooperative NOMA networks. *IEEE Commun. Lett.* **23**, 760–763 (2019).

[8] Khan, M. J., Chauhan, R. C. S., Singh, I., Fatima, Z. & Singh, G. Mobility management in heterogeneous network of vehicular communication with 5G: Current status and future perspectives. *IEEE Access* **12**, 86271–86292 (2024).

[9] Huang, L., Zhu, B., Nan, R., Chi, K. & Wu, Y. Attention-based SIC ordering and power allocation for non-orthogonal multiple access networks. *IEEE Trans. Mobile Comput.* (2024).

[10] Xu, P., Yang, Z., Ding, Z. & Zhang, Z. Optimal relay selection schemes for cooperative NOMA. *IEEE Trans. Veh. Technol.* **67**, 7851–7855 (2018).

[11] Yue, X. et al. Simultaneously transmitting and reflecting reconfigurable intelligent surface assisted NOMA networks. *IEEE Trans. Wireless Commun.* **22**, 189–204 (2022).

[12] Samy, M., Al-Hraishawi, H., Chatzinotas, S. & Otteresten, B. Outage performance of multiple hybrid active relays and RISs-assisted NOMA networks. *IEEE Wirel. Commun. Lett.* **13**, 2322–2326 (2024).

[13] Aldababsa, M., Khaleel, A. & Basar, E. Simultaneous transmitting and reflecting reconfigurable intelligent surfaces-empowered NOMA networks. *IEEE Syst. J.* **17**, 5441–5451 (2023).

[14] Zhao, B., Zhang, C., Yi, W. & Liu, Y. Ergodic rate analysis of STAR-RIS aided NOMA systems. *IEEE Commun. Lett.* **26**, 2297–2301 (2022).

[15] Yang, S., Ding, Z. & Zhu, H. STAR-RIS aided multi-antenna NOMA downlink and uplink transmissions: A low-complexity approach. *IEEE Trans. Wireless Commun.* **23**, 10773–10787 (2024).

[16] Salem, A., Wong, K.-K., Chae, C.-B. & Zhang, Y. STAR-RIS assisted full-duplex NOMA communication networks. *IEEE Trans. Wireless Commun.* **24**, 2467–2482 (2025).

[17] Yue, X. et al. Active simultaneously transmitting and reflecting surface assisted NOMA networks. *IEEE Trans. Wireless Commun.* **23**, 9912–9926 (2024).

[18] Yue, X. et al. ASTARS aided NOMA covert communication networks. *IEEE Trans. Veh. Technol.* **74**, 10661–10673 (2025).

[19] Yue, X. et al. Federated learning in ASTARS aided uplink networks. *IEEE Trans. Veh. Technol.* **75**, 1156–1170 (2025).

[20] Tien Ban, N. & Le, H.-C. Transmission probabilities of adaptive RIS-aided NOMA systems over Fisher–Snedecor-F channels. *IEEE Open J. Commun. Soc.* **6**, 8460–8476 (2025).

[21] Khalil, M., Wang, K. & Choi, J. Adaptive power allocation in spaceborne assisted NOMA systems for integrated terrestrial communications. arXiv preprint arXiv:2410.11254 (2024).

[22] Vishwakarma, L. K., Gour, R., Yadav, S. & Silva, A. STAR-RIS-NOMA empowered vehicle-to-vehicle communications: Outage and ergodic capacity analysis. *Veh. Commun.* **50**, 100852 (2024).

[23] Tang, S. et al. STAR-RIS assisted reliable and secure transmissions in wireless-powered communications. *IEEE Trans. Wireless Commun.* **23**, 19836–19851 (2024).

[24] Singh, A., Singh, K., Kaushik, A., Ng, D. W. K. & Biswas, S. Adaptive neural network optimization for fully connected STAR-RIS-aided NOMA networks. In *2025 IEEE International Conference on Communications Workshops (ICC Workshops)* 190–195 (IEEE, 2025).

[25] Iqbal, M. et al. A comprehensive survey on reconfigurable intelligent surfaces (RIS) and STAR-RIS for next-generation wireless networks. *Discov. Appl. Sci.* **7**, 1253 (2025).

[26] Jeffrey, A. & Zwillinger, D. *Table of Integrals, Series, and Products* (Elsevier Science, 2007).

[27] Tang, P. et al. STAR-RIS-aided AAV-NOMA networks: Joint resource allocation and trajectory optimization. *IEEE Trans. Green Commun. Netw.* **10**, 134–148 (2026).

[28] Liu, J. Energy-harvesting relay-assisted STAR-RIS-enhanced vehicular NOMA networks. *Veh. Commun.* 100960 (2025).

[29] Yang, S., Zhang, J., Xia, W., Gao, H. & Zhu, H. Joint power and discrete amplitude allocation for STAR-RIS-aided NOMA system. *IEEE Trans. Veh. Technol.* **71**, 13382–13386 (2022).

[30] Prudnikov, A., Brychkov, I. & Marichev, O. *Integrals and Series*, vol. 3 of *Integrals and Series* (Gordon and Breach Science Publishers, 1986).

[31] Kumar, D., Singh, C. K., López, O. L. A., Bhatia, V. & Latva-aho, M. Performance analysis of active RIS-assisted downlink NOMA with transmit antenna selection. *IEEE Trans. Veh. Technol.* **74**, 7774–7791 (2025).

[32] Abromowitz, M. & Stegun, I. A. Handbook of Mathematical Functions (1972).

[33] Toregozhin, T., Shaikh, M. H. N., Almagambetov, A. & Nauryzbayev, G. Performance of STAR-RIS-aided cooperative NOMA networks under Nakagami-m fading. *Ad Hoc Netw.* **156**, 103399 (2024).

[34] Ahmed, M. et al. A survey on STAR-RIS: Use cases, recent advances, and future research challenges. *IEEE Internet Things J.* **10**, 14689–14711 (2023).

